# Hippocampal volume changes after (*R,S*)-ketamine administration in patients with major depressive disorder and healthy volunteers

**DOI:** 10.1038/s41598-024-54370-9

**Published:** 2024-02-24

**Authors:** Jennifer W. Evans, Morgan C. Graves, Allison C. Nugent, Carlos A. Zarate

**Affiliations:** 1 Experimental Therapeutics and Pathophysiology Branch, National Institute of Mental Health, National Institutes of Health, 10 Center Dr., Bldg 10, Rm 7-3335, Bethesda, MD 20814 USA; 2 MEG Core, NIMH, Bethesda, MD USA

**Keywords:** Magnetic resonance imaging, Translational research, Depression

## Abstract

The hippocampus and amygdala have been implicated in the pathophysiology and treatment of major depressive disorder (MDD). Preclinical models suggest that stress-related changes in these regions can be reversed by antidepressants, including ketamine. Clinical studies have identified reduced volumes in MDD that are thought to be potentiated by early life stress and worsened by repeated depressive episodes. This study used 3T and 7T structural magnetic resonance imaging data to examine longitudinal changes in hippocampal and amygdalar subfield volumes associated with ketamine treatment. Data were drawn from a previous double-blind, placebo-controlled, crossover trial of healthy volunteers (HVs) unmedicated individuals with treatment-resistant depression (TRD) (3T: 18 HV, 26 TRD, 7T: 17 HV, 30 TRD) who were scanned at baseline and twice following either a 40 min IV ketamine (0.5 mg/kg) or saline infusion (acute: 1–2 days, interim: 9–10 days post infusion). No baseline differences were noted between the two groups. At 10 days post-infusion, a slight increase was observed between ketamine and placebo scans in whole left amygdalar volume in individuals with TRD. No other differences were found between individuals with TRD and HVs at either field strength. These findings shed light on the timing of ketamine’s effects on cortical structures.

## Introduction

Changes in the hippocampus^1,2^ and amygdala^3,4^ have been implicated in the pathophysiology and treatment of major depressive disorder (MDD)^5^. A recent meta-analysis of individuals with MDD found an approximate reduction of 8% in bilateral hippocampal volume and a smaller reduction in the amygdala (~ 7% on the right and ~ 5% on the left). It should be noted, however, that although studies have consistently documented hippocampal volume reductions in depressed patients, these differences have typically been small (Cohen’s d = − 0.14)^6^. Furthermore, other studies found no differences in overall hippocampal volume between healthy volunteers (HVs) and MDD patients despite standardizing the definition of the structures by improving automated segmentation^7–9^. These mixed results have spurred investigations to examine changes in subfield volumes that may be more sensitive to specific regional changes.

Reduced hippocampal volumes have been reported in rodent models in relation to stress-related reductions in neural plasticity^5^. Hippocampal subareas CA1, after 2 weeks, and CA3 and the dentate gyrus and subiculum after 4 weeks^10^ seem to be particularly affected supporting the idea that subfield volume changes may be useful to examine rather than overall volume. Similar to the findings in rodents, chronic stress in MDD patients has been linked to atrophy of apical dendrites in the CA1 and CA3 subfields as well as decreased neurogenesis in the dentate gyrus^11–14^. Smaller amygdalar volumes have also been linked to recent stressful life events in depressed patients and HVs^14^, though findings from the magnetic resonance imaging (MRI) volumetric literature are variable with regard to differences in the overall size of the amygdala between depressed patients and HVs^3^. Most of this work has been done at 3T and, interestingly, recent work with higher resolution scans at 7T with automatic segmentation of the amygdala and hippocampal subfields have revealed associations between volumetric size and severity of depressive symptoms^15^.

Preclinical models have also suggested that some of the stress-related reductions in plasticity associated with MDD can be reversed by antidepressants, including the rapid-acting glutamatergic modulator ketamine^5,16^. For instance, prior rodent studies found that in vivo ketamine administration enhanced dendritic spine density and restored dendritic spine loss in the medial prefrontal cortex^17–19^. Directly complementary human studies to investigate the effects of antipressant changes on the brain cannot yet be pursued as no direct in vivo measurement of plasticity or dendritic spine density in humans is possible^20^. However, structural changes in volumetric magnetic resonance imaging (MRI) have been found to be partially explained by increases in dendritic spine density^21^ and also clinically useful to investigate brain changes in response to antidepressants^22^.

Ketamine’s rapid mechanism of action, where symptom relief occurs within hours and days instead of weeks or months, makes it ideal for assessing whether its antidepressant effects measurably alter hippocampal or amygdalar volume over the course of treatment. Interestingly, a single infusion of racemic (*R,S*)-ketamine (hereafter referred to as ketamine) was previously found to reduce the volume of the left nucleus accumbens but increase the volume of the left hippocampus in MDD patients who achieved remission following treatment^23^. Another recent MRI study found that *S*-ketamine—the *S* enantiomer of ketamine—altered hippocampal volume as soon as 65 min after a single infusion^24^. These results echo previous findings that antidepressant treatments and electroconvulsive therapy (ECT) both cause volumetric increases in these structures^25^. It is also important to note that, while the link between amygdalar volume changes and antidepressant response is less clear, functional studies found that ketamine alters amygdalar response^26^ and connectivity^27^ in both HVs and individuals with MDD.

This study used both 3T and 7T structural MRI data to examine longitudinal changes in hippocampal and amygdalar subfield volumes post-ketamine infusion (0.5 mg/kg administered over 40 min) at baseline. Scan were acquired at acute (1–2 days, or maximum symptom improvement) and interim (9–10 days, where symptoms are returning) timepoints. Data were drawn from a previous double-blind, placebo-controlled, crossover trial of HVs and unmedicated patients with treatment-resistant depression (TRD) who nominally participated in all 10 scans across both field strengths. The goals of this study were to evaluate (1) whether the effects of a therapeutic dose of ketamine produce changes in hippocampal and/or amydalar volumes at the chosen timepoints (2) reliability of segmentations in our longitudinal sample at and between each field strength. In line with previous findings, the hypothesis was that smaller hippocampal and amygdalar volumes would be observed at baseline in individuals with TRD and that ketamine treatment would increase these volumes. This unique dataset with repeated measurements in the same individuals was also used to evaluate the stability of our subfield segmentations, longitudinally and across field strengths, which will provide continuity between past and current investigations of these structures.

## Methods

### Participants and study design

These data were collected as part of a randomized, double-blind, placebo-controlled, crossover, single-site experimental study (NCT00088699, NIH Protocol 04-M-0222); results have previously been published^28^. Thirty-two unique TRD participants and 21 unique HVs were included in this analysis. All participants were between the ages of 18–65 years old and were required to sign written and informed consent before enrolling in the study as approved by the National Institutes of Health (NIH) Combined Central Nervous System Institutional Review Board and in accordance with the Declaration of Helsinki. TRD participants fulfilled DSM-IV criteria for recurrent MDD without psychotic features based on clinical assessment and confirmed by a structural diagnostic interview (SCID); MADRS scores were ≥ 20 at screening and before ketamine or saline infusion. In addition, TRD participants’ current depressive episode had lasted at least 4 weeks, and they had not responded to at least one antidepressant medication during their current major depressive episode. TRD participants were tapered off psychotropic medicationsover a 1- to 2-weeks period, if necessary, and were free of any psychotropic medications for at least 2 weeks prior to the first infusion (the taper period was extended to 3 weeks for aripiprazole and 5 weeks for fluoxetine which equates to five half-lives (The amount of drug remaining after a half life is N/2, where N is the original quantity. After 4 half-lives the remaining amount of drug is (1/2)^4^ = 6.25%, and similarly after 5 half-lives is 3.125%, of the original amount. As 94–97% of the drug is eliminated after 4–5 half-lives, the remaining amount is considered below clinical relevance 29^29^.) of the respective drugs).

HVs had no previous psychiatric history of a current or past DSM-IV Axis I diagnosis. Exclusion criteria included psychiatric disorders in their first-degree relatives or any medical condition that alters brain morphology and/or physiology, including those controlled by medication.

All participants were randomized to first receive an infusion of either 0.5 mg/kg ketamine hydrochloride or a saline solution over 40 min. In order to avoid any carry-over effects between infusion sessions, participants who completed the first arm were blindly crossed over to receive the other treatment following a 2 weeks interval. No psychotherapy or pharmaceutical intervention was permitted during the entirety of the study.

**Figure 1:**
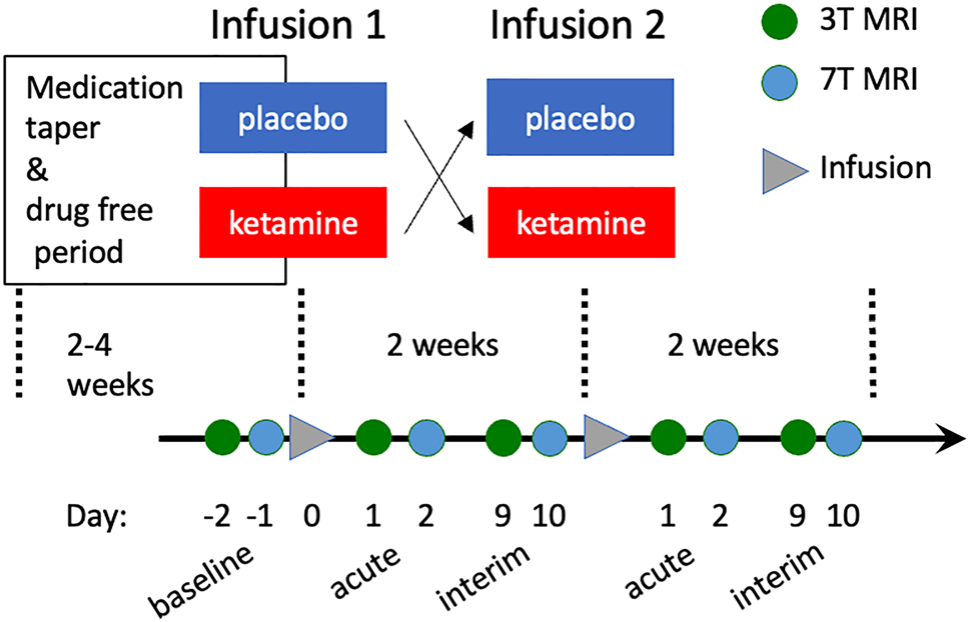
Study design illustrating timing of 3T and 7T scans with respect to the infusions.

MRI scans were conducted at baseline (1 to 2 days before the first infusion) and at acute and interim time points after each infusion (about 1 and 9 days for 7T scans and about 2 and 10 days for 3T, respectively), for a total of 10 scans per participant as shown in Fig. 1. Scans were nominally acquired at the same time of day. Psychometric ratings, as assessed via the MADRS^30^, were clinician-administered 60 min before both ketamine and placebo infusions as well as on the imaging days.

### Volumetric analysis

7T scans were performed on a Siemens Magnetom (Erlangen, Germany) scanner using a 32-channel head coil (Nova Medical, Wilimington, MA). High resolution T_1_ weighted MPRAGE images were acquired (256 slices; 0.7 mm isotropic resolution; repetition time (TR): 2200 ms; echo time (TE): 3.01 ms; field of view (FOV): 224 mm; flip angle (FA): 7°; inversion time: 1050 ms) and spatially matched proton density weighted (256 slices; 0.7 mm isotropic resolution; TR: 1470 ms; TE: 3.01 ms; FOV: 224 mm; FA: 10°).

3T scans were performed on a GE HDx (Milwaukee, WI) scanner with the system eight-channel head coil. The parameters for the T_1_ weighted scans were: 3D fast spoiled gradient recalled echo (FSPGR) sequence; TR: 8.8 s; TE: 3.4 ms; inversion recovery time (IR): 450 ms; FA: 13°; 1mm isotropic resolution.

#### Skull stripping for 7T scans

Skull stripping was performed prior to segmentation using two different methods: OptiBET^31^ and Multiple-cONtrast brain STRipping method (MONSTR)^32^ (see supplementary methods for the commands used.). The skull stripped results were examined visually, and the best brain extraction was used as the input to the segmentation algorithm.

#### Hippocampus and amygdala segmentation

Hippocampal segmentation was performed using FreeSurfer v. 6.0^33^ (Martinos Center for Biomedical Imaging, Charlestown, MA, USA) separately for the 3T and 7T scans (see supplementary methods for the commands used). Briefly, the set of images across all time points per participant was processed through longitudinal hippocampal and amygdalar subfield segmentation pipelines^34–36^. The longitudinal pipelines used unbiased template volumes created for each participant^37^, which were used as initial approximations for cortical and subcortical segmentation of the white matter and deep gray matter volumetric structures. Initializing the preprocessing steps (skull stripping, Talairach transforms, atlas registration, spherical surface maps and parcellations*)* using common information from the within-subject template significantly increases reliability and statistical power^38^. An example of the segmentation into 19 subfields for the hippocampus and nine for the amygdala for a single, randomly chosen participant, is shown in Supplementary Fig. S1.

### Statistical and stability analysis

Statistical analysis was performed in R (4.3.1) using separate mixed linear effects models on the hippocampal and amygdalar subfield volumes (see supplementary materials for all libraries and versions used along with detailed model descriptions). Separate models were used to evaluate baseline data and longitudinal changes across scans for hippocampal and amygdalar subfield volumes at each field strength. Common covariates included factors of sex (male, female) and diagnosis (HV, TRD) as well as continuous variables of body mass index (BMI) and eTIV. Age, eTIV, and sex are included as recommended covariates to use in the case of volume estimation^39^. Diagnosis was included because it is part of the research question being examined and a defining feature between our groups. Similarly, BMI has also been implicated as affecting brain volume^40^. Scan timepoint (acute, interim) was also included as a factor in the longitudinal model. In line with current guidelines for reporting statistical significance, CIs were reported at the 95% level throughout, along with precise raw *p* values^41^ and also an adjusted *p* value corrected for multiple comparisons using the Hochberg method using p.adjust in R.

Within-field strength subfield measurement stability was evaluated by calculating percent change from baseline for volumetric change, the Dice coefficient of overlap, and ICC for measurement reliability. Dice coefficients of overlap between the subfield segmentations from repeated scans for each participant were calculated using 3dSliceNDice from the FATCAT toolbox^42^. ICCs were calculated in R with ICC (ICC3k fixed raters means) from the psych package. Percent difference from baseline was calculated by subtracting the subfield volume at each scan timepoint from baseline, dividing by baseline volume, and multiplying by 100%. Finally, between-field strength volume comparisons were performed using Bland–Altman analysis^43^ for the whole amygdala and hippocampus for for participants with both scans at baseline (HV: 15, MDD: 21).

## Results

### Demographics

Demographic information, including Montgomery-Asberg Depression Rating Scale (MADRS) scores, for the individuals included in the 3T and 7T analyses can be found in Table 1. Although the same participants were scanned at both field strengths, some scans were not acquired for scheduling reasons, resulting in a different number of participants across field strengths. In addition, five participants (two with TRD and three HVs) were excluded from the 7T data image processing due to data quality issues.

**Table 1:** Demographics for healthy volunteers (HVs) and individuals with treatment-resistant depression (TRD) showing mean values and standard error in brackets.

		3T		7T	
		HV (N = 18)	TRD (N = 26)	HV (N = 17)	TRD (N = 30)
Gender					
F		12 (66.7%)	16 (61.5%)	10 (58.8%)	17 (56.7%)
M		6 (33.3%)	10 (38.5%)	7 (41.2%)	13 (43.3%)
Age					
Mean (SD)		33.7 (11.0)	34.0 (8.40)	34.0 (11.1)	33.9 (8.54)
Median [Min, Max]		30.5 [20.0, 56.0]	32.0 [20.0, 55.0]	29.0 [20.0, 56.0]	32.0 [20.0, 55.0]
BMI					
Mean (SD)		28.1 (4.33)	28.4 (7.39)	28.2 (4.25)	27.2 (5.94)
Median [Min, Max]		27.8 [20.9, 34.7]	26.7 [19.7, 50.4]	27.5 [21.9, 34.7]	25.5 [19.7, 44.5]
eTIV					
Mean (SD)		1,560,000 (160,000)	1,540,000 (161,000)	1,220,000 (333,000)	1,150,000 (226,000)
Median [Min, Max]		1,520,000 [1320000, 1880000]	1,550,000 [1200000, 1920000]	1,140,000 [830000, 1740000]	1,080,000 [754000, 1650000]
Age of onset					
Mean (SD)			15.3 (7.11)		15.0 (5.79)
Median [Min, Max]			13.0 [4.00, 33.0]		15.0 [4.00, 31.0]
Number of previous episodes					
Mean (SD)			2.58 (2.27)		2.30 (2.74)
Median [Min, Max]			1.00 [1.00, 9.00]		1.00 [0, 9.00]
MADRS					
Baseline (mean, 95% CI)		1.6 [1, 2.4]	31.6 [29, 34.1]	1.6 [1, 2.4]	33.3 [31, 35.2]
Acute Acute	Ketamine Placebo	1 [0, 1.7] 0.1 [− 0, 0.3]	29.4 [26, 32.7] 32.7 [30, 35.0]	0.9 [0, 1.6] 0.2 [− 0, 0.4]	27.7 [24, 31.6] 33.1 [31, 35.6]
Interim Interim	Ketamine Placebo	1.7 [1, 2.9] 0.7 [0, 1.3]	23.3 [19, 27.4] 31 [29, 33.4]	1.9 [− 0, 3.9] 0.2 [− 0, 0.5]	25.2 [21, 29.5] 31.2 [28, 34.1]

*HV* healthy volunteer, *TRD* treatment-resistant depression, *BMI* body mass index, *eTIV* estimated total intracranial volume, *MADRS* montgomery-asberg depression rating scale.

### Baseline whole hippocampal/amygdalaand subfield differences

**Figure 2:**
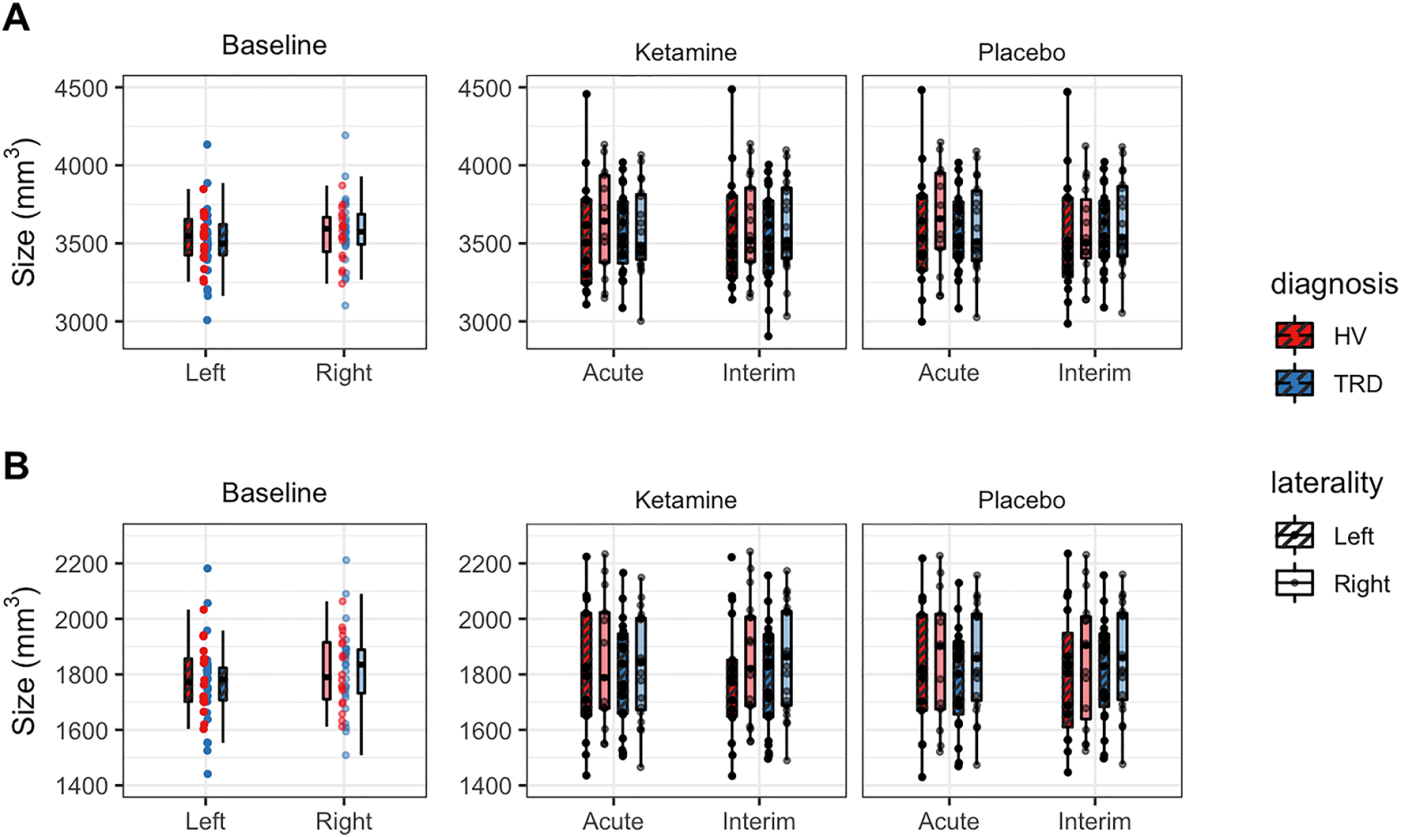
Box-whisker plots illustrating the (**A**) hippocampal (top) and (**B**) whole amygdalar (bottom) volumes at 3T for healthy volunteers (HVs) (red) and individuals with treatment-resistant depression (TRD) (blue) at baseline, acute, and interim scans after ketamine and placebo infusions. Dots indicate volume for individual participants; the boxplot illustrates the mean and quartiles of the distributions.

For the 3T scans, whole hippocampal and amygdalar left and right hemisphere volumes for the HV and TRD groups at baseline are shown in the box-whisker plots in Fig. 2A; corresponding quantitative values can be found in Supplementary Table S1. For the 7T scans, values and plots can be found in Supplementary Table S2 and Fig. S2A. At 3T, the mixed model at baseline revealed a main effect of sex for the left (confidence interval (CI) [29, 344], F_1,33_ = 5.6 *p* = 0.02) and right hippocampus (CI [95, 302], F_1,33_ = 4.55, *p* = 0.04) and for the right amygdala (CI [114, 325], F_1,33_ = 18.0, *p* < 0.001) (see Supplementary Table 3 for volumes for each sex). At 7T, a main effect was observed for estimated total intracranial volume (eTIV) (CI [0.002, 0.001], F_5,38_ = 3.9 *p* = 0.004) for the left and right hippocampus (CI [0.002, 0.001], F_5,38_ = 4.3, *p* = 0.001) as well as for the right (CI [0.0004,0.0006], F_5,38_ = 4.3, *p* = 0.01) and left amygdala (CI [0.0001,0.0007], F_5,38_ = 4.3, *p* = 0.03).

No differences were found between groups for any of the subfield regions at baseline for either field strength; see Supplementary Tables S4, S5 for mean values and statistical values between groups and Supplementary Figs. S3, S4 for subfield volumes.

### Longitudinal differences: effect of ketamine

No changes in whole hippocampal volumes were found at either acute or interim post-ketamine scans or after placebo administration for either hemisphere or group or field strength (Fig. 2A for 3T and Fig. S2A for 7T). At 3T, the left amygdala showed an increase of 36.61 mm^3^ (95 %CI  [7, 66] mm^3^, F_1,89_ = 2.44, *p* = 0.02) for the TRD group at the acute scan between ketamine and placebo conditions. No other changes in amygdalar volume were found for any other group, hemisphere scan, or field strength (Fig. 2B for 3T and Fig. S2B for 7T).

Average amygdalar values at 3T for the TRD and HV groups are illustrated in the box-whiskers plots in Fig. 2B (see Fig. S2B for 7T) for the acute and interim scans for both ketamine and placebo infusions. Quantitative differences between the acute and interim scans for both the TRD and HV groups are shown in Table 2. Any subfield changes noted between ketamine and placebo infusions at either acute or interim scans did not survive multiple comparisons testing (Supplementary Table S6).

**Table 2:** Difference between ketamine and placebo whole hippocampal and amygdalar volumes for healthy volunteers (HVs) and individuals with treatment-resistant depression (TRD) at acute and interim scans at 3T (main effect of drug for each timepoint).

Region	Diagnosis	Interval	Estimate	Est.CI	t.ratio	*p* value	Adjusted.p
L_Whole_amygdala	HV	Acute	5.84	[-30, 41]	0.32	0.75	0.88
L_Whole_amygdala	TRD	Acute	36.61	[7, 66]	2.44	0.02	0.26
L_Whole_amygdala	HV	Interim	− 12.87	[− 45, 19]	− 0.78	0.44	0.75
L_Whole_amygdala	TRD	Interim	− 0.81	[− 30, 28]	− 0.06	0.96	0.96
L_Whole_hippocampus	HV	Acute	− 25.95	[− 63, 11]	− 1.36	0.18	0.75
L_Whole_hippocampus	TRD	Acute	2.47	[− 28, 33]	0.16	0.88	0.93
L_Whole_hippocampus	HV	Interim	17.07	[− 17, 51]	0.99	0.33	0.75
L_Whole_hippocampus	TRD	Interim	− 18.16	[− 48, 12]	− 1.17	0.24	0.75
R_Whole_amygdala	HV	Acute	5.84	[− 33, 45]	0.29	0.77	0.88
R_Whole_amygdala	TRD	Acute	− 8.22	[− 40, 24]	− 0.50	0.62	0.82
R_Whole_amygdala	HV	Interim	11.66	[− 23, 47]	0.65	0.52	0.75
R_Whole_amygdala	TRD	Interim	13.32	[− 18, 45]	0.83	0.41	0.75
R_Whole_hippocampus	HV	Acute	− 15.02	[− 56, 26]	− 0.72	0.47	0.75
R_Whole_hippocampus	TRD	Acute	− 23.44	[− 57, 10]	− 1.37	0.17	0.75
R_Whole_hippocampus	HV	Interim	14.24	[− 23, 51]	0.76	0.45	0.75
R_Whole_hippocampus	TRD	Interim	− 20.43	[− 53, 12]	− 1.22	0.23	0.75

### Within scanner stability: percent difference from baseline across subfields

**Figure 3:**
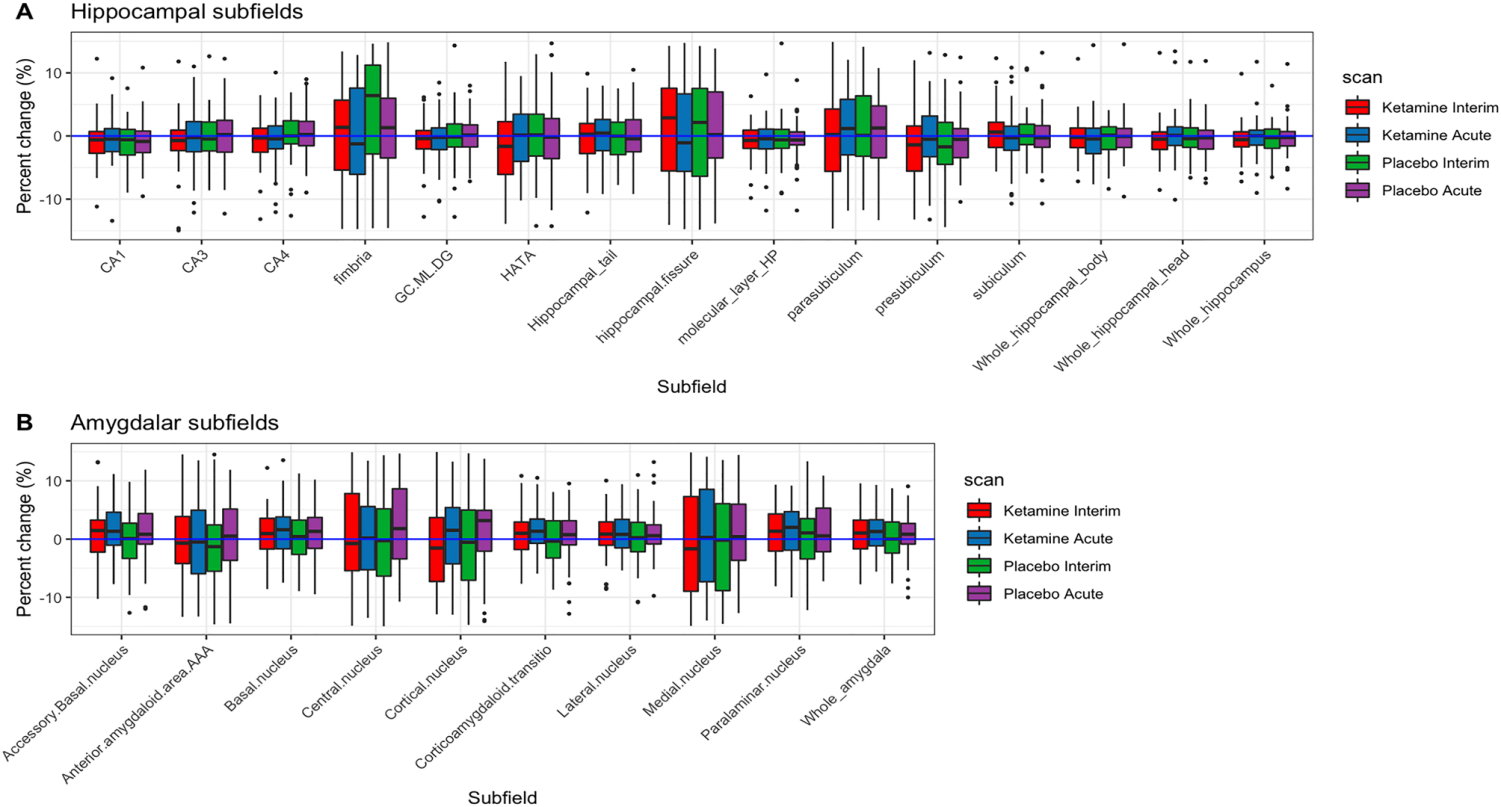
Subfield volume differences from baseline at acute and interim scans after ketamine and placebo infusions for (**A**) hippocampus and (**B**) amygdala at 3T. Abbreviations: *HATA* hippocampal-amygdaloid transition region; *GC ML DG* granule cells in the molecular layer of the dentate gyrus; *CA1-4* cornu ammonis.

The percent change in volume of hippocampal and amygdalar subfields measured between baseline and post-infusion scans across all participants at 3T is shown in Fig. 3 (see Supplementary Fig. S5 for 7T data). Supplementary Table S7 presents the differences between acute and baseline scans. For the hippocampus, the greatest stability in percent change between scans was observed for the whole hippocampus (mean difference = − 0.048%, SE = 0.15) and subiculum (mean difference = − 0.122%, SE = 0.20), whereas the fimbria (mean difference = 2.6%, SE = 0.64) and hippocampal fissure (mean difference = 2.9%, SE = 0.60) showed the least stability. For the amygdala, both the whole amygdala and its subfields were in general more variable than those of the hippocampus. The anterior amygdaloid area (mean difference = 2.9%, SE = 0.60) and lateral nucleus (mean difference = 1.1%, SE = 0.52) were the most consistent, and the central (mean difference = 3.8%, SE = 0.72) and medial (mean difference = 5.7%, SE = 1.2) nuclei were the least consistent. The amount of variability in percent difference between scans was consistent for a given subfield.

### Dice coefficient and ICC

The calculated Dice coefficient overlap and ICC for all subfields across all sessions and participants indicated that the subfields with the highest Dice coefficient were the basal (0.92, CI [0.86, 0.96]) and lateral nuclei (0.92, CI [0.86, 0.95]) of the amygdala; the medial (0.29, CI [0.05, 0.57]) and paralaminar nuclei (0.55, CI [0.32, 0.73]) had the lowest Dice values (Supplementary Table S8). Typically, 7T subfield segmentations had slightly higher Dice values than their 3T counterparts with the exception of the molecular layer. ICC values across scans were uniformly very high for all subfields, with most being above 0.99, showing excellent agreement of volume measurement between scans; the medial nucleus, with an ICC of 0.96, CI [0.95,0.97], was an exception.

### Comparison of 3T and 7T volumes

**Figure 4:**
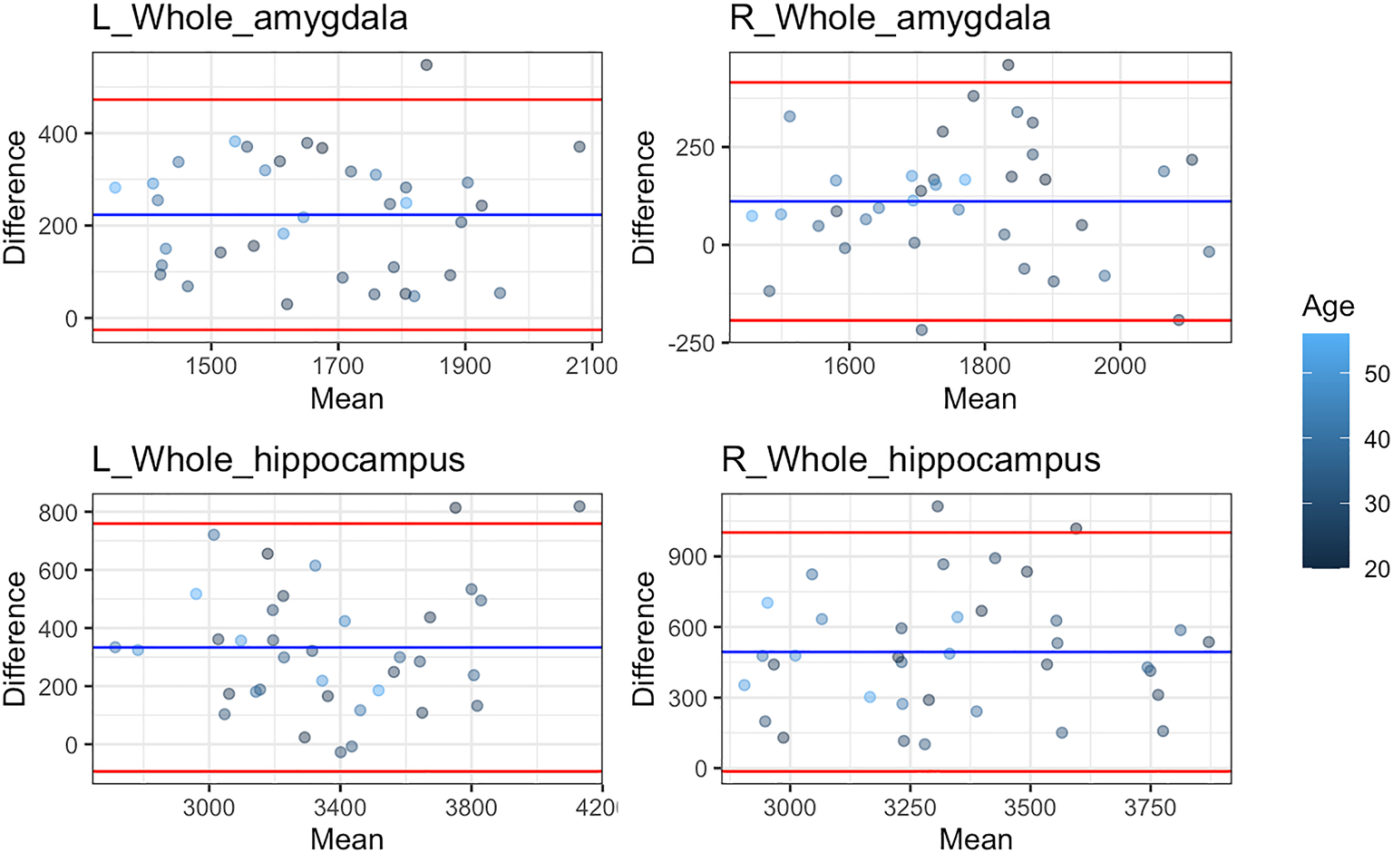
Bland-Altman^43^ plot of total gray matter measured at baseline between 3 and 7T scans within the same individuals for bilateral whole amygdala and hippocampus. Each dot represents the difference between 3 and 7T total gray matter for an individual plotted against the mean of total gray matter across both field strengths for that same individual for the region specified in the plot title. The red lines are plotted at one standard deviation of the differences and the blue line represents the overall mean of the differences. The dots are coloured by the age of the participant.

The Bland-Altman^43^ plot illustrates the difference and mean between 3 and 7T whole hippocampal and amygdalar volumes estimated for participants with scans at both field strengths at baseline (Fig. 4). 3T volumes tended to be larger than those estimated with 7T, with the exception of the right amygdala, where a few participants had larger 7T estimates. Overall this volumetric difference was 145 mm^3^ CI [93, 198] for the right and 257 mm^3^ CI  [214, 301] for the left whole amygdala, 528 mm^3^ CI [441, 616] for the right whole hippocampus and 367 mm^3^ CI [294, 441] for the left.

## Discussion

This study used repeated 3T and 7T imaging in the same individuals to investigate (a) longitudinal differences between TRD patients and HVs during a double-blind, placebo-controlled ketamine trial and (b) measurement reliability in hippocampal and amygdalar subfield volumes between the scanners at different field strengths. No differences in total hippocampal volume were found between individuals with TRD and HVs at baseline or at any point during the study. A measurable increase in whole left amygdalar volume was observed in TRD patients between ketamine and placebo at the post-infusion acute scan (approximately 2 days). No other differences in whole amygdalar volumes were found between individuals with TRD and HVs. The few differences in hippocampal and amygdalar subfield volumes post-ketamine did not survive multiple comparisons correction. Within field strength reliability was best in whole hippocampus and amygdala rather than subfield segmentations. 7T segmentations had better Dice metric overlap than 3T but ICC values were uniformly high across all subfields at both field strengths. On average volume estimates at 3T were slightly larger than the estimates at 7T.

Many reports in the literature of meta-anlyses have foundreduced hippocampal volumes in individuals with MDD compared to HVs^1,6,44–49^. More recent studies, however, have yielded a mix of positive and negative results for both whole volumes and subfields. Some factors that may account for the mixed findings reported in the literature include differences in length of illness, the age at onset of depressive episodes, patient history of trauma, and the heterogeneity of the MDD samples, which might include a mix of medicated, unmedicated, and medication-naïve individuals. The current patient sample was completely unmedicated but not medication-naïve as their medications had been tapered just prior to the study. Medication for depression has been shown to provide a neuroprotective effect where greater hippocampal volume decreases occur from lack of treatment^50^. Other studies have more inhomogeneity in the medication status of their participants which could suggest that current or past medication use plays a role in hippocampal volume.

For instance, Brown and colleagues found reduced hippocampal subfields at 7T in a sample of medication-free MDD patients with a similar sample size but where only about half of their patients had TRD or were unmedicated. Further, Roddy and colleagues found that a group of MDD patients who had experienced repeated depressive episodes had significantly decreased bilateral hippocampal subfield volumes. 73% of their patients were medicated which also. However, Phillips and colleagues also found no difference between MDD patients and HVs at baseline, though they did find that longitudinal structural trajectories differed in patients depending on their clinical response to treatment after 6 months, with non-remitters exhibiting smaller hippocampi at that timepoint^51^. An exploratory analysis investigating the association of volume change after ketamine and MADRS response did not show a significant relation (Fig. S6). Similarly, there was no significant correlation between baseline volumes and initial MADRS score for our patients (Fig. S7). Echoing previous preclinical findings, Abdallah and colleagues found that ketamine had enhanced antidepressant effects in MDD patients with smaller hippocampal volumes at baseline In contrast, our results echo those of Kraus and colleagues, who found no change in hippocampal volume after treatment with selective serotonin reuptake inhibitors (SSRIs) after 12 weeks of treatment Our scans were acquired around 2 and 10 days post-ketamine infusion to capture the acute response and return to baseline changes of symptoms that occur after the initial metabolism of the drug. As we do not measure a volumetric change at these distal time points, ketamine’s primary effects on synaptogenesis may occur within a shorter period of time suggesting the possibility that hippocampal volume change may occur early after an infusion and that this, potentially transient, change may not be correlated with sustained symptom improvement. However, a recent paper found that six repeated doses of ketamine administered in conjunction with conventional antidepressants increased both amygdalar and hippocampal volumes Asthis study did not measure changes after a single dose, it is unclear whether or not the measured volumetric changes accumulated over time or were apparent immediately after the first ketamine treatment and maintained by subsequent doses. Regardless, these findings point to the fact that medication status should be controlled for in future studies.

On a more technical note, previous differences in hippocampal volume changes have been attributed to disparate hippocampal definitions or different segmentation algorithms^52^. In this context, it should be noted that most new studies use automatic segmentation, which improves the comparability of studies. Our reliability results closely resemble those previously reported in healthy adults^53,54^ and support the reliability of Freesurfer segmentations at both field strengths. High ICC test–retest values in hippocampal subfields were found in this study comparable to others that used the longitudinal pipeline^54,55^. The repeated measurements in this study align well to the intent of Freesurfer’s longitudinal pipeline which provides robust subfield segmentations even for the challenging 7T data. One drawback of this possibly improved consistency is that segmentation stability may obscure individual differences, and that more individualized segmentation algorithms may yield better results^52^. New algorithmic developments using deep learning^56^ have been proposed which may further improve the accuracy of hippocampal segmentation.

Based on our measurements, a minimum change of 40-50mm^3^ would be required to be detected in the amygdala and 70–100 mm^3^ in the hippocampus at 3T (and for amygdala 80–90 mm^3^ and in hippocampus 130–150 mm^3^ at 7T). Despite the within field strength stability there are notable difference in estimated volumes between them. This may be partially due to the difference in resolutions of the acquisition as it is possible, and standard, to acquire higher resolution data at higher field strengths due to the increased signal at 7T. Thus, standardized acquisition parameters are likely required, in addition to standardized pipelines to increase reproducibility and interoperability between studies.

Limitations of the present study include the small sample size, though this was somewhat mitigated by the longitudinal cross-over study design with repeated scans at two field strengths Though the study was randomized and double-blinded, the blinding may have been imperfect and could have affected the subjective response because ketamine has psychomimetic effects^57^. A way to mitigate this issue is to assess expectations for the dissociative experience using a validated tool^58^ and incorporate it into analyses. In addition, there is a difference in group sizes which could reduce our sensitivity to detect between group changes which is mitigated by our use of mixed-models will help account for the ‘missing data’ in between group comparisons. The study also used MRI measurements of anatomical volumes, which are limited by the base resolution of the image acquisition (typically around 1 mm) and rely on several factors that could not be controlled for in this study (e.g., hydration, motion). Future studies could consider controlling for such factors and others such as time of day. Nevertheless, breaking the hippocampus into its constituent subfields is more consistent with how research has been conducted in animals (i.e., targeting one type of cell response). This study was designed to investigate the acute and immediate effects of a single ketamine infusion; it is possible that repeated ketamine doses, which newer studies have found to be associated with volumetric changes in the hippocampus, might yield different results. Despite these limitations, additional strengths of the study include that the patient sample was diagnosed with TRD and had been tapered off all psychotropic medications. Additionally, the same participants were scanned at both field strengths and repeatedly on the same scanners which provides a unique baseline characterization for the analysis pipeline used here.

In conclusion, this study—which used data from the same participants scanned at both 3T and 7T resolutions—found that in individuals with TRD, a single, acute ketamine infusion did not affect the size of the hippocampus or its subfields during the time period measured in this study. Furthermore, no significant baseline differences were observed between HVs and TRD patients. Underlying hippocampal and amygdalar impairment in TRD patients may occur below the threshold (amygdala, 3T: 40–50 mm^3^, 7T: 80–90 mm^3^; hippocampus, 3T: 70–100 mm^3^, 7T: 130–150 mm^3^) of our ability to measure volumetric changes here. Research to improve the sensitivity and consistency of theseinvestigational techniques is warranted.

### Supplementary Information

Supplementary Information.

## Data Availability

The data that support the findings of this study are available from the corresponding author upon request. The data are not publicly available due to privacy or ethical restrictions.
