# Supplementary material for: Hippocampal volume changes after (R,S)-ketamine administration in patients with major depressive disorder and healthy volunteers
Source: Sci Rep. 2024 Feb 24;14:4538. doi: 10.1038/s41598-024-54370-9 (PMC10894199; doi:10.1038/s41598-024-54370-9)
Supplement: Supplementary file 1 — Supplementary Information. [file 41598_2024_54370_MOESM1_ESM.docx]

*Evans et al — Hippocampal volume changes after (R,S)-ketamine administration in*

*patients with major depressive disorder and healthy volunteers*

**Supplementary Materials**

**Supplementary Methods**

Skullstripping commands

OptiBET - <https://montilab.psych.ucla.edu/fmri-wiki/optibet/>

opti-bet.sh -i T1.nii.gz

where T1.gz was a T_1_-weighted anatomical scan.

MONSTR - <https://www.nitrc.org/projects/monstr>

MONSTR.sh --t1 T1w.nii --pd PD.nii --ncpu 4 --natlas 5 --robust --clean --o output_dir

Segmentation commands

Freesurfer 6: <https://surfer.nmr.mgh.harvard.edu/fswiki/rel6downloads>

Longitudinal pipeline:

<https://surfer.nmr.mgh.harvard.edu/fswiki/LongitudinalProcessing>

recon-all -subjid sub -sd output_dir -i T1w.nii.gz -autorecon-all -openmp 1

recon-all -base sub_sesb -tp ses1 -tp ses2 -tp ses3 -tp ses 4 -all

recon-all -long -subjid sub_sesb -sd output_dir -i T1w.nii.gz -autorecon-all -openmp 1

hippocampal/amydalar segmentation: <https://surfer.nmr.mgh.harvard.edu/fswiki/LongitudinalHippocampalSubfields>

segmentHA_T1_long.sh sub

Configuration for R:

R version 4.3.1 (2023-06-16)

Platform: aarch64-apple-darwin20 (64-bit)

Running under: macOS Ventura 13.5

Matrix products: default

BLAS: /System/Library/Frameworks/Accelerate.framework/Versions/A/Frameworks/vecLib.framework/Versions/A/libBLAS.dylib

LAPACK: /Library/Frameworks/R.framework/Versions/4.3-arm64/Resources/lib/libRlapack.dylib; LAPACK version 3.11.0

locale:

[1] en_US.UTF-8/en_US.UTF-8/en_US.UTF-8/C/en_US.UTF-8/en_US.UTF-8

time zone: America/New_York

tzcode source: internal

attached base packages:

[1] stats graphics grDevices utils datasets methods base

other attached packages:

[1] ggpmisc_0.5.4-1 ggpp_0.5.4 tableone_0.13.2 patchwork_1.1.3

[5] RColorBrewer_1.1-3 nlme_3.1-162 reshape2_1.4.4 emmeans_1.8.8

[9] car_3.1-2 carData_3.0-5 ggpattern_1.0.1 ggplot2_3.4.3

[13] broom_1.0.5 table1_1.4.3 purrr_1.0.2 tidyr_1.3.0

[17] stringr_1.5.0 stringi_1.7.12 kableExtra_1.3.4 knitr_1.43

[21] dplyr_1.1.3

Statistical models

*Baseline whole hippocampal/amygdala and subfield differences*

region volume ~ BMI + Age + Diagnosis + Sex + eTIV + (1|subject)

- Diagnosis is a between-subject factor with two levels (TRD, HV)
- Sex is a within-subject factor with two levels (male, female)
- BMI, body mass index, is a continuous variable
- Age, is a continuous variable
- eTIV, estimated total intracranial volume is a continuous variable
- (1|subject) is a random effect within subject

Region is one of the bilateral (right or left): whole hippocampus, whole amygdala or one of the hippocampal (19) or amygdalar (9) subfields listed below. There are a total or 30 regions of interest.

Hippocampal subfields

Parasubiculum

HATA

Fimbria

Hippocampal fissure

HP_tail

Presubiculum-head

Presubiculum-body

Subiculum-head

Subiculum-body

CA1-head

CA1-body

CA3-head

CA3-body

CA4-head

CA4-body

GC-ML-DG-head

GC-ML-DB-body

Molecular_layer_HP-head

Molecular_layer_HP-body

Amygdalar subfields

Lateral nucleus

Basal nucleus

Central nucleus

Medial nucleus

Cortical nucleus

Accessory Basal nucleus

Cortioamygdaloid transition

Anterior amygdaloid area

Paralaminar nucleus

*Effect of ketamine*

Region volume ~ BMI + eTIV + Age + Sex + Diagnosis*Drug*Interval + (1|subject),

- Diagnosis is a between-subject factor with two levels (TRD, HV)
- Sex is a within-subject factor with two levels (male, female)
- Drug is a within-subject factor with two levels (ketamine, placebo)
- Interval is a within-subject factor with two levels (acute, interim)
- BMI, body mass index, is a continuous variable
- Age, is a continuous variable
- eTIV, estimated total intracranial volume is a continuous variable
- (1|subject) is a random effect within subject

**Supplementary Figures**

Supplementary Figure S1. Hippocampal and amygdalar subfield segmentation example from one representative participant with labels.


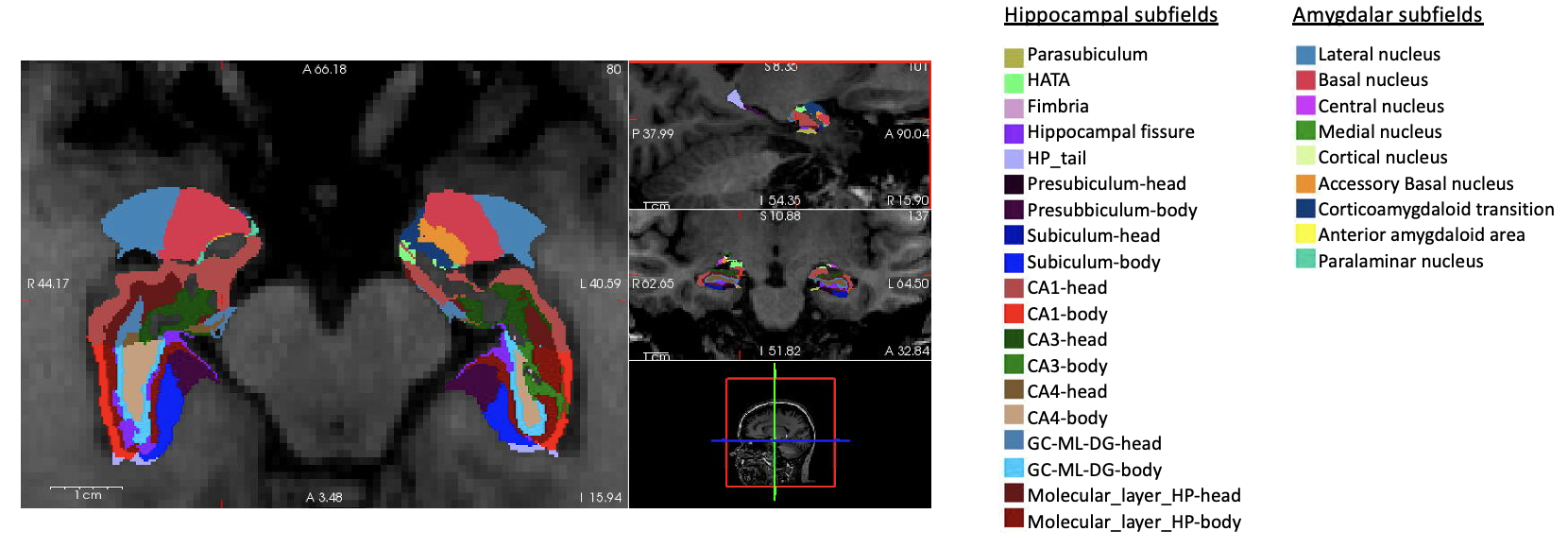


CA: Cornu Ammonis; GC: granule cell; ML: molecular layer; DG: dentate gyrus; HP: hippocampus; HATA: hippocampal amygdala transition area

Supplementary Figure S2. 7T A) hippocampal and B) amygdalar volume estimates
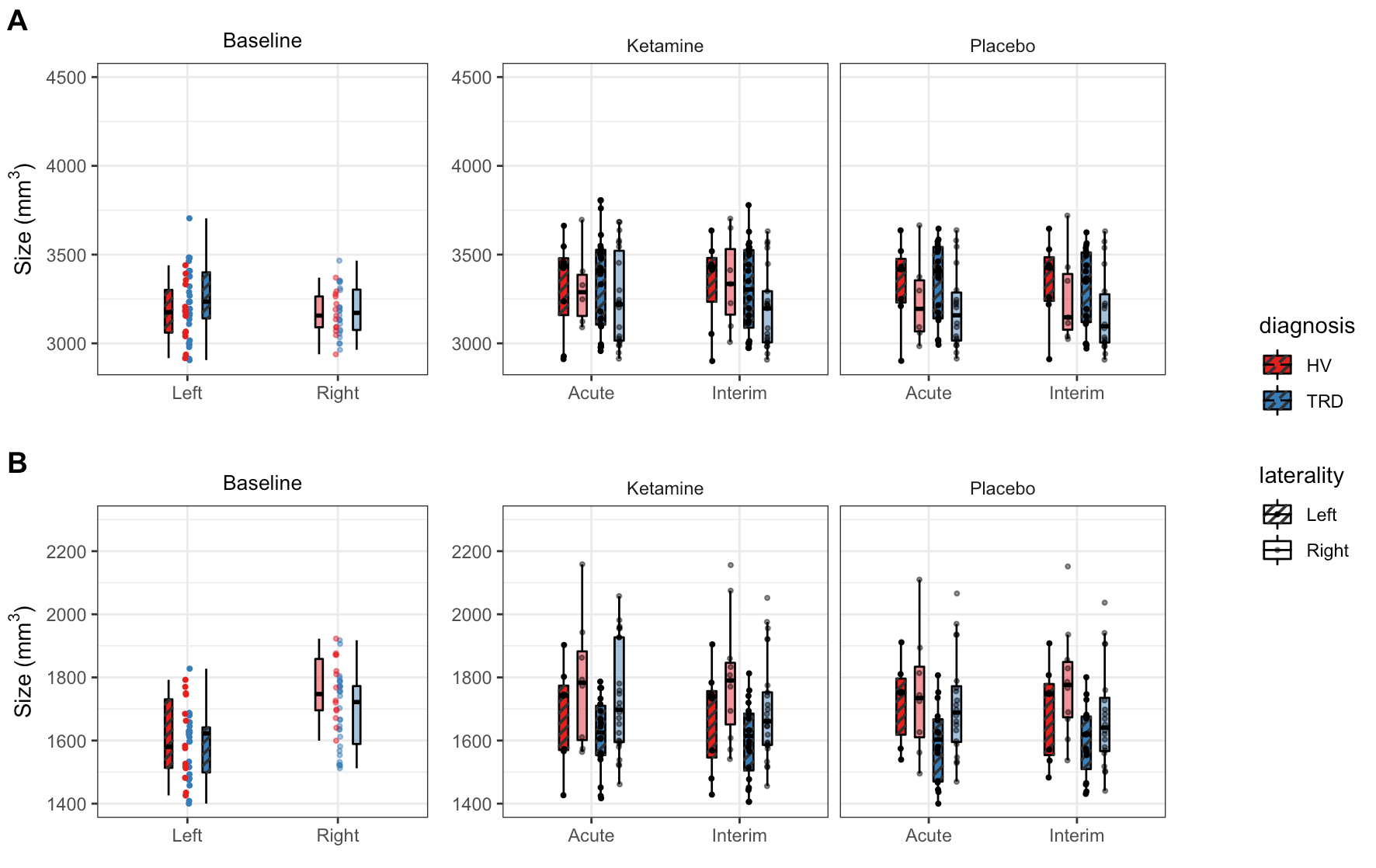


Supplementary Figure S3. Subfield volumes across hemisphere and diagnosis at 3T


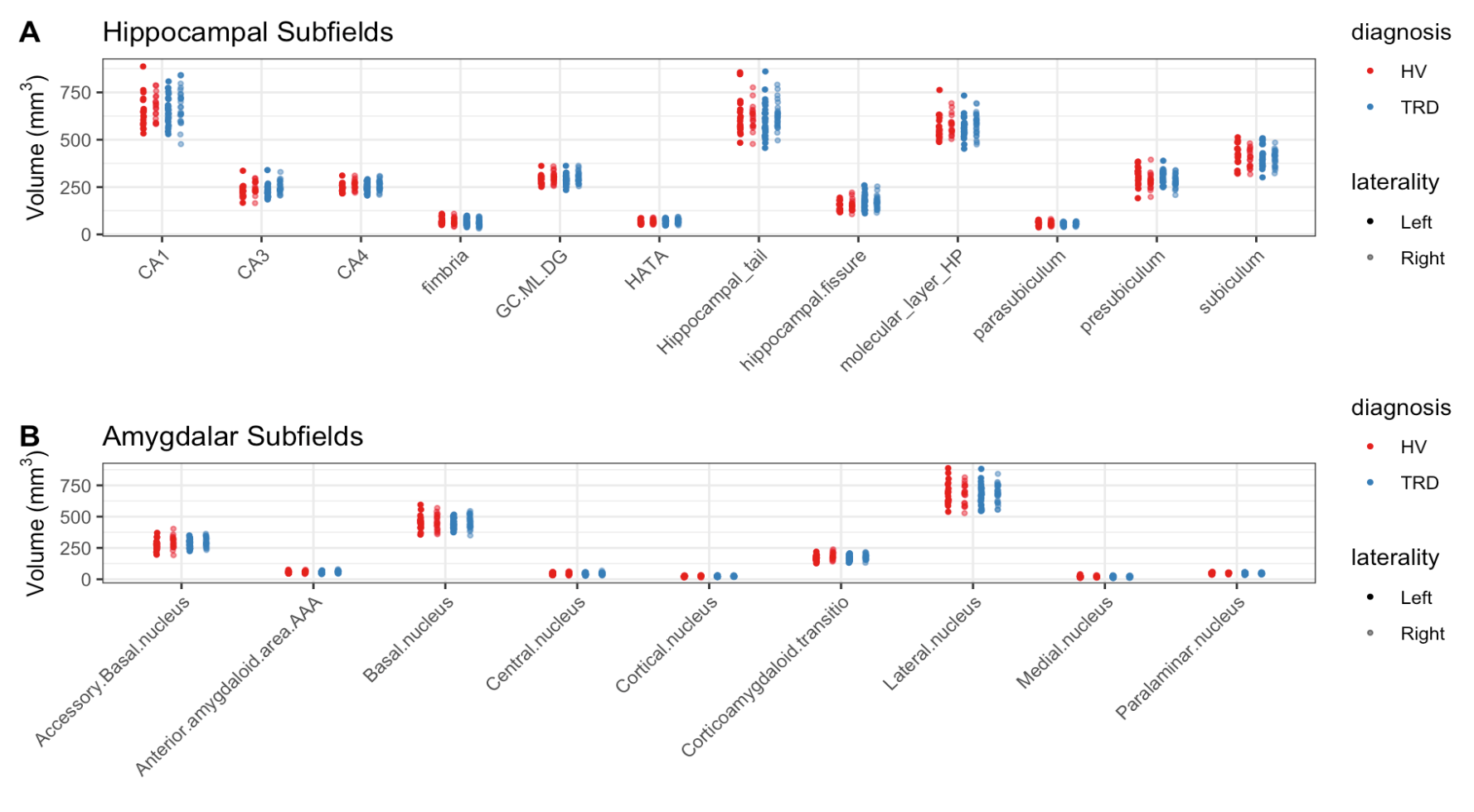


Supplementary Figure S4. Subfield volumes across hemisphere and diagnosis at 7T


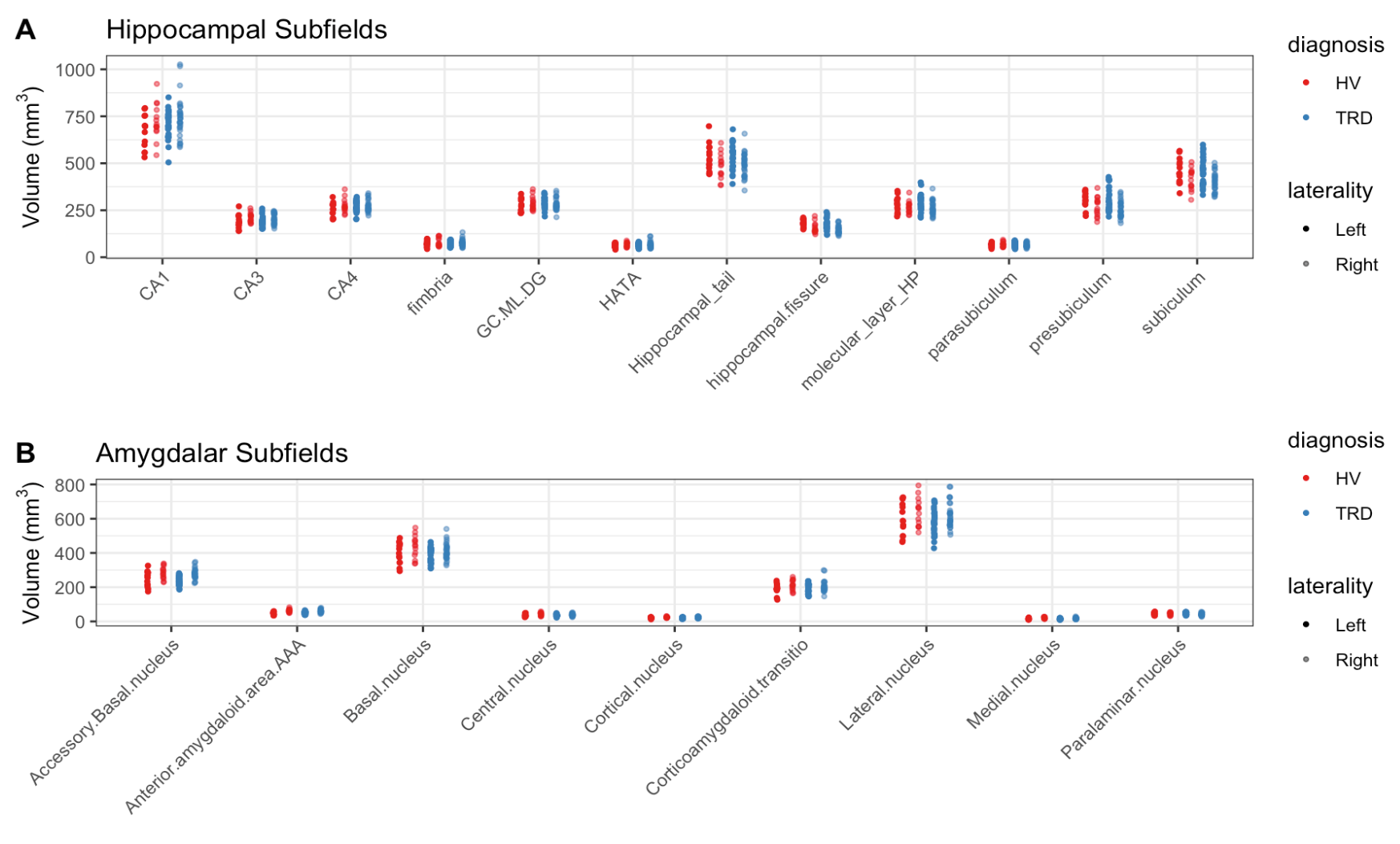


Supplementary Figure S5. Subfield volume differences from baseline at acute and interim scans after ketamine and placebo infusions for A) hippocampus and B) amygdala at 7T
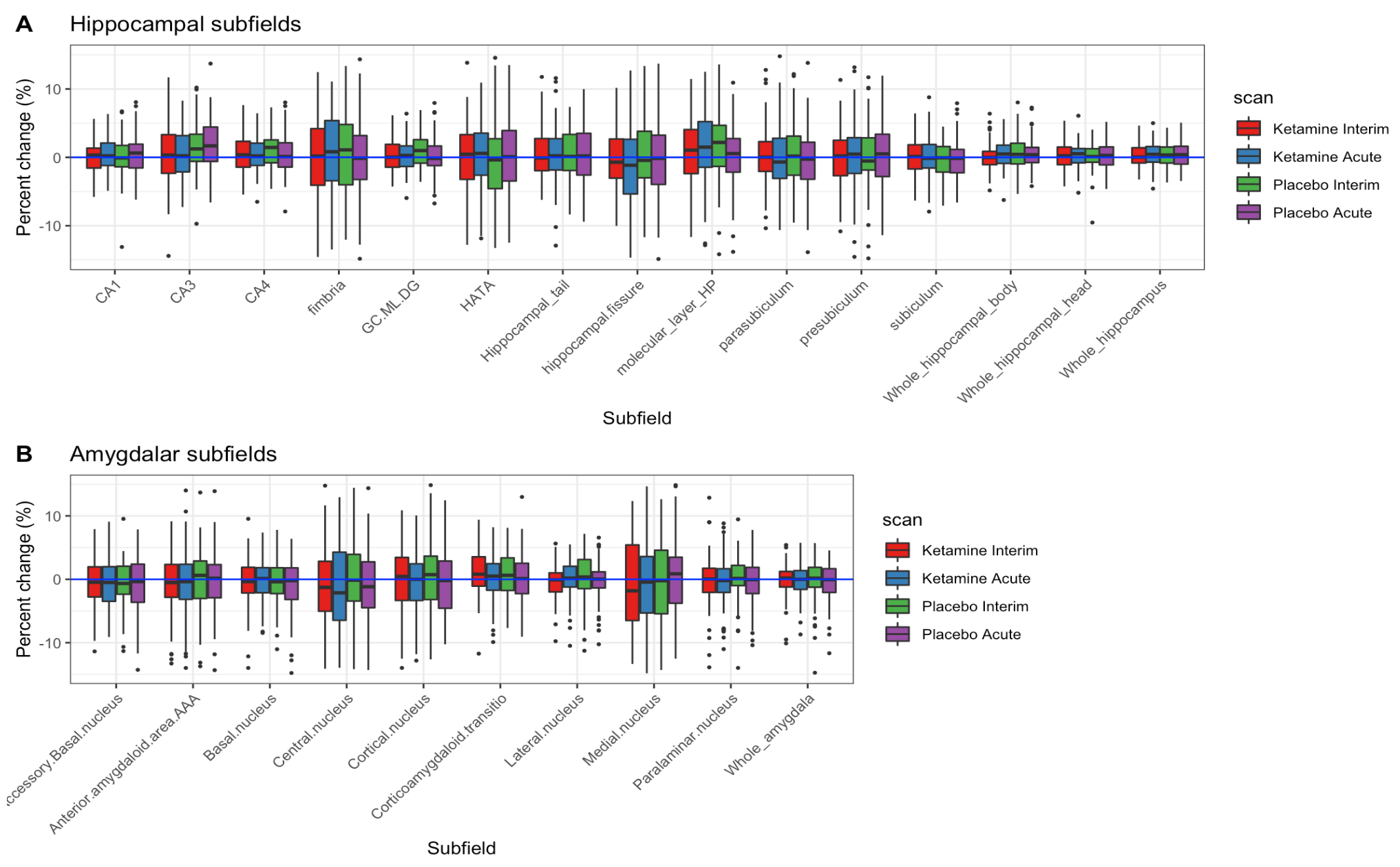


**Figure S6. Association of baseline volume at 3T with percent change from baseline measured 2 days after ketamine.** A linear fit of the values is displayed (blue line) along with the 95% confidence interval of the fit (gray shading. The equation of best fit is also displayed along with the goodness of fit (R^2^).

**Figure S7. Association of MADRS with baseline volumes at 3T.** A linear fit of the values is displayed (blue line) along with the 95% confidence interval of the fit (gray shading. The equation of best fit is also displayed along with the goodness of fit (R^2^).


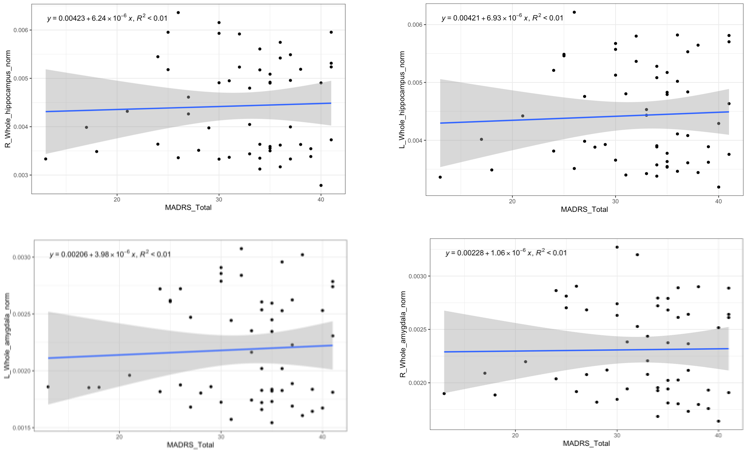


**Supplementary Tables**

Supplementary Table S1. Mean whole hippocampal and amygdala volumes for healthy volunteers (HV) and individuals with treatment-resistant depression (TRD) at baseline at 3T

| **Region** | **HV**  **Mean** | **95%CI** | **TRD**  **Mean** | **95%CI** | **estimate** | **95%CI** | **p.value** | **adjusted.p** |
| --- | --- | --- | --- | --- | --- | --- | --- | --- |
| L_Whole_amygdala | 1814.65 | [ 1745 , 1885 ] | 1794.39 | [ 1737 , 1852 ] | -20.26 | [ -109 , 69 ] | 0.66 | 0.95 |
| L_Whole_hippocampus | 3547.82 | [ 3417 , 3679 ] | 3532.15 | [ 3424 , 3640 ] | -15.67 | [ -182 , 150 ] | 0.85 | 0.95 |
| R_Whole_amygdala | 1841.56 | [ 1772 , 1911 ] | 1844.15 | [ 1787 , 1902 ] | 2.60 | [ -86 , 91 ] | 0.95 | 0.95 |
| R_Whole_hippocampus | 3575.26 | [ 3464 , 3687 ] | 3604.73 | [ 3513 , 3696 ] | 29.47 | [ -112 , 170 ] | 0.68 | 0.95 |
| L= left, R = Right |  |  |  |  |  |  |  |  |

Supplementary Table S2. Mean whole hippocampal and amygdala volumes for healthy volunteers (HV) and individuals with treatment-resistant depression (TRD) at baseline at 7T

| Region | HV.Mean | 95% CI | TRD.Mean | 95% CI | estimate | 95% CI | p.value | **adjusted.p** |
| --- | --- | --- | --- | --- | --- | --- | --- | --- |
| L_Whole_amygdala | 1611.62 | [ 1533 , 1691 ] | 1583.20 | [ 1528 , 1638 ] | -28.42 | [ -124 , 67 ] | 0.56 | 0.97 |
| L_Whole_hippocampus | 3171.28 | [ 3022 , 3321 ] | 3275.30 | [ 3172 , 3379 ] | 104.02 | [ -77 , 285 ] | 0.27 | 0.95 |
| R_Whole_amygdala | 1765.32 | [ 1680 , 1851 ] | 1712.00 | [ 1653 , 1771 ] | -53.32 | [ -157 , 50 ] | 0.32 | 0.95 |
| R_Whole_hippocampus | 3138.60 | [ 2998 , 3279 ] | 3135.18 | [ 3038 , 3233 ] | -3.42 | [ -174 , 167 ] | 0.97 | 0.97 |

L= left, R = Right

Suplementary Table S3. Estimated mean whole hippocampal and amygdala volumes across sex at 3T.

|  | Sex | Volume (mm^3^) | SE | df | lower.CL | upper.CL |
| --- | --- | --- | --- | --- | --- | --- |
| L_Whole_amygdala | F | 3453.05 | 54.00 | 38 | 3343.72 | 3562.37 |
|  | M | 3626.93 | 73.42 | 38 | 3478.30 | 3775.56 |
| L_Whole_hippocampus | F | 3515.51 | 45.85 | 38 | 3422.70 | 3608.32 |
|  | M | 3664.48 | 62.33 | 38 | 3538.30 | 3790.65 |
| R_Whole_amygdala | F | 1705.66 | 28.86 | 38 | 1647.23 | 1764.09 |
|  | M | 1903.38 | 39.24 | 38 | 1823.95 | 1982.82 |
| R_Whole_hippocampus | F | 1738.52 | 28.75 | 38 | 1680.33 | 1796.72 |
|  | M | 1947.19 | 39.08 | 38 | 1868.07 | 2026.30 |

L= left, R = Right, F= female, M=male

Supplementary Table S4. Baseline Subfield Volumes and group differences at 3T.

| **Region** | **HV.Mean** | **HV.CI** | **TRD.Mean** | **TRD.CI** | **estimate** | **est.CI** | **p.value** | **adjusted.p** |  |  |
| --- | --- | --- | --- | --- | --- | --- | --- | --- | --- | --- |
| L_Accessory.Basal.nucleus | 281.18 | [ 266 , 296 ] | 289.69 | [ 277 , 302 ] | 8.51 | [ -11 , 28 ] | 0.39 | 0.99 |  |  |
| L_amygNucleus | 4197.13 | [ 4058 , 4336 ] | 4305.63 | [ 4191 , 4420 ] | 108.51 | [ -68 , 285 ] | 0.24 | 0.99 |  |  |
| L_Anterior.amygdaloid.area | 56.92 | [ 54 , 60 ] | 55.88 | [ 54 , 58 ] | -1.04 | [ -5 , 3 ] | 0.58 | 0.99 |  |  |
| L_Basal.nucleus | 461.77 | [ 444 , 480 ] | 454.00 | [ 439 , 469 ] | -7.77 | [ -30 , 15 ] | 0.50 | 0.99 |  |  |
| L_CA1 | 654.86 | [ 621 , 689 ] | 660.68 | [ 633 , 689 ] | 5.82 | [ -37 , 49 ] | 0.79 | 0.99 |  |  |
| L_CA3 | 231.27 | [ 217 , 246 ] | 233.84 | [ 222 , 246 ] | 2.57 | [ -16 , 21 ] | 0.78 | 0.99 |  |  |
| L_CA4 | 251.48 | [ 242 , 261 ] | 250.44 | [ 243 , 258 ] | -1.04 | [ -13 , 11 ] | 0.86 | 0.99 |  |  |
| L_Central.nucleus | 45.31 | [ 42 , 48 ] | 43.06 | [ 40 , 46 ] | -2.24 | [ -6 , 2 ] | 0.28 | 0.99 |  |  |
| L_Cortical.nucleus | 23.05 | [ 21 , 25 ] | 23.69 | [ 22 , 25 ] | 0.63 | [ -1 , 3 ] | 0.55 | 0.99 |  |  |
| L_Corticoamygdaloid.transition | 174.30 | [ 167 , 182 ] | 174.88 | [ 169 , 181 ] | 0.58 | [ -9 , 10 ] | 0.91 | 0.99 |  |  |
| L_fimbria | 74.36 | [ 68 , 81 ] | 68.47 | [ 63 , 74 ] | -5.89 | [ -14 , 2 ] | 0.16 | 0.99 |  |  |
| L_GC.ML.DG | 289.78 | [ 279 , 300 ] | 289.59 | [ 281 , 298 ] | -0.19 | [ -13 , 13 ] | 0.98 | 0.99 |  |  |
| L_HATA | 66.77 | [ 63 , 71 ] | 68.17 | [ 65 , 72 ] | 1.39 | [ -4 , 7 ] | 0.61 | 0.99 |  |  |
| L_Hippocampal_tail | 622.05 | [ 578 , 666 ] | 609.12 | [ 573 , 645 ] | -12.93 | [ -68 , 42 ] | 0.65 | 0.99 |  |  |
| L_hippocampal.fissure | 148.05 | [ 133 , 164 ] | 171.72 | [ 159 , 185 ] | 23.67 | [ 4 , 43 ] | 0.02 | 0.99 |  |  |
| L_HippoSf | 4197.13 | [ 4058 , 4336 ] | 4305.63 | [ 4191 , 4420 ] | 108.51 | [ -68 , 285 ] | 0.24 | 0.99 |  |  |
| L_Lateral.nucleus | 701.63 | [ 667 , 736 ] | 686.28 | [ 658 , 714 ] | -15.35 | [ -59 , 28 ] | 0.49 | 0.99 |  |  |
| L_Medial.nucleus | 20.96 | [ 18 , 24 ] | 18.85 | [ 17 , 21 ] | -2.11 | [ -5 , 1 ] | 0.22 | 0.99 |  |  |
| L_molecular_layer_HP | 566.35 | [ 540 , 592 ] | 572.51 | [ 551 , 594 ] | 6.15 | [ -27 , 39 ] | 0.72 | 0.99 |  |  |
| L_Paralaminar.nucleus | 49.54 | [ 47 , 52 ] | 48.07 | [ 46 , 50 ] | -1.47 | [ -4 , 1 ] | 0.32 | 0.99 |  |  |
| L_parasubiculum | 59.44 | [ 55 , 63 ] | 55.53 | [ 52 , 59 ] | -3.91 | [ -9 , 1 ] | 0.14 | 0.99 |  |  |
| L_presubiculum | 309.44 | [ 291 , 328 ] | 304.16 | [ 289 , 319 ] | -5.28 | [ -29 , 18 ] | 0.66 | 0.99 |  |  |
| L_subiculum | 422.01 | [ 396 , 448 ] | 419.63 | [ 399 , 441 ] | -2.38 | [ -35 , 30 ] | 0.89 | 0.99 |  |  |
| L_Whole_amygdala | 1814.65 | [ 1745 , 1885 ] | 1794.39 | [ 1737 , 1852 ] | -20.26 | [ -109 , 69 ] | 0.66 | 0.99 |  |  |
| L_Whole_hippocampal_body | 1219.84 | [ 1173 , 1266 ] | 1219.78 | [ 1181 , 1258 ] | -0.06 | [ -59 , 59 ] | 1.00 | 0.99 |  |  |
| L_Whole_hippocampal_head | 1705.94 | [ 1636 , 1776 ] | 1703.25 | [ 1645 , 1761 ] | -2.69 | [ -92 , 86 ] | 0.95 | 0.99 |  |  |
| L_Whole_hippocampus | 3547.82 | [ 3417 , 3679 ] | 3532.15 | [ 3424 , 3640 ] | -15.67 | [ -182 , 150 ] | 0.85 | 0.99 |  |  |
| R_Accessory.Basal.nucleus | 301.46 | [ 286 , 317 ] | 302.29 | [ 290 , 315 ] | 0.83 | [ -19 , 20 ] | 0.93 | 0.99 |  |  |
| R_amygNuc | 4420.76 | [ 4280 , 4561 ] | 4494.44 | [ 4378 , 4610 ] | 73.68 | [ -105 , 252 ] | 0.42 | 0.99 |  |  |
| R_Anterior.amygdaloid.area | 59.76 | [ 56 , 63 ] | 61.09 | [ 58 , 64 ] | 1.34 | [ -3 , 6 ] | 0.56 | 0.99 |  |  |
| R_Basal.nucleus | 463.31 | [ 444 , 483 ] | 466.63 | [ 450 , 483 ] | 3.33 | [ -22 , 28 ] | 0.79 | 0.99 |  |  |
| R_CA1 | 674.66 | [ 641 , 708 ] | 689.11 | [ 662 , 717 ] | 14.44 | [ -28 , 57 ] | 0.51 | 0.99 |  |  |
| R_CA3 | 249.28 | [ 236 , 262 ] | 251.60 | [ 241 , 262 ] | 2.32 | [ -14 , 19 ] | 0.78 | 0.99 |  |  |
| R_CA4 | 263.03 | [ 253 , 273 ] | 260.63 | [ 252 , 269 ] | -2.40 | [ -15 , 11 ] | 0.72 | 0.99 |  |  |
| R_Central.nucleus | 46.41 | [ 42 , 51 ] | 46.19 | [ 43 , 50 ] | -0.22 | [ -6 , 5 ] | 0.94 | 0.99 |  |  |
| R_Cortical.nucleus | 25.56 | [ 24 , 27 ] | 24.48 | [ 23 , 26 ] | -1.07 | [ -3 , 1 ] | 0.35 | 0.99 |  |  |
| R_Corticoamygdaloid.transition | 188.70 | [ 181 , 196 ] | 181.95 | [ 176 , 188 ] | -6.75 | [ -17 , 3 ] | 0.18 | 0.99 |  |  |
| R_fimbria | 71.28 | [ 64 , 79 ] | 64.28 | [ 58 , 70 ] | -7.00 | [ -16 , 2 ] | 0.15 | 0.99 |  |  |
| R_GC.ML.DG | 303.35 | [ 293 , 314 ] | 301.96 | [ 293 , 311 ] | -1.39 | [ -15 , 12 ] | 0.84 | 0.99 |  |  |
| R_HATA | 69.40 | [ 65 , 73 ] | 71.45 | [ 68 , 75 ] | 2.05 | [ -3 , 7 ] | 0.44 | 0.99 |  |  |
| R_Hippocampal_tail | 623.30 | [ 595 , 652 ] | 634.71 | [ 611 , 658 ] | 11.41 | [ -25 , 48 ] | 0.54 | 0.99 |  |  |
| R_hippocampal.fissure | 155.71 | [ 142 , 169 ] | 170.07 | [ 159 , 181 ] | 14.36 | [ -3 , 32 ] | 0.11 | 0.99 |  |  |
| R_HippoSf | 4420.76 | [ 4280 , 4561 ] | 4494.44 | [ 4378 , 4610 ] | 73.68 | [ -105 , 252 ] | 0.42 | 0.99 |  |  |
| R_Lateral.nucleus | 687.30 | [ 659 , 716 ] | 694.98 | [ 672 , 718 ] | 7.67 | [ -28 , 44 ] | 0.68 | 0.99 |  |  |
| R_Medial.nucleus | 20.77 | [ 18 , 23 ] | 19.72 | [ 18 , 22 ] | -1.05 | [ -4 , 2 ] | 0.53 | 0.42 |  |  |
| R_molecular_layer_HP | 580.49 | [ 557 , 604 ] | 584.16 | [ 565 , 603 ] | 3.67 | [ -26 , 33 ] | 0.81 | 0.99 |  |  |
| R_Paralaminar.nucleus | 48.29 | [ 46 , 50 ] | 46.81 | [ 45 , 49 ] | -1.48 | [ -4 , 1 ] | 0.29 | 0.99 |  |  |
| R_parasubiculum | 57.65 | [ 53 , 62 ] | 57.00 | [ 53 , 61 ] | -0.65 | [ -6 , 5 ] | 0.82 | 0.99 |  |  |
| R_presubiculum | 282.51 | [ 265 , 300 ] | 283.44 | [ 269 , 298 ] | 0.93 | [ -21 , 23 ] | 0.94 | 0.99 |  |  |
| R_subiculum | 400.30 | [ 381 , 420 ] | 406.38 | [ 390 , 423 ] | 6.09 | [ -19 , 31 ] | 0.63 | 0.99 |  |  |
| R_Whole_amygdala | 1841.56 | [ 1772 , 1911 ] | 1844.15 | [ 1787 , 1902 ] | 2.60 | [ -86 , 91 ] | 0.95 | 0.99 |  |  |
| R_Whole_hippocampal_body | 1223.21 | [ 1175 , 1271 ] | 1224.83 | [ 1185 , 1264 ] | 1.62 | [ -59 , 62 ] | 0.96 | 0.99 |  |  |
| R_Whole_hippocampal_head | 1728.75 | [ 1665 , 1793 ] | 1745.19 | [ 1692 , 1798 ] | 16.44 | [ -65 , 98 ] | 0.69 | 0.99 |  |  |
| R_Whole_hippocampus | 3575.26 | [ 3464 , 3687 ] | 3604.73 | [ 3513 , 3696 ] | 29.47 | [ -112 , 170 ] | 0.68 | 0.99 |  |  |

Abbreviations: CA: Cornu Ammonis; GC: granule cell; ML: molecular layer; DG: dentate gyrus; HP: hippocampus; HATA: hippocampal amygdala transition area.

Supplementary Table S5. Baseline Subfield Volumes and group differences at 7T

| **Region** | **HV.Mean** | **HV.CI** | **TRD.Mean** | **TRD.CI** | **estimate** | **est.CI** | **statistic** | **p.value** |  | **adjusted.p** |
| --- | --- | --- | --- | --- | --- | --- | --- | --- | --- | --- |
| L_Accessory.Basal.nucleus | 248.71 | [ 235 , 262 ] | 242.25 | [ 233 , 252 ] | -6.46 | [ -23 , 10 ] | -0.76 | 0.45 |  | 0.99 |
| L_amygNuc | 3653.55 | [ 3471 , 3836 ] | 3672.81 | [ 3547 , 3799 ] | 19.26 | [ -201 , 240 ] | 0.17 | 0.87 |  | 0.99 |
| L_Anterior.amygdaloid.area.AAA | 47.26 | [ 44 , 51 ] | 50.10 | [ 48 , 53 ] | 2.85 | [ -1 , 7 ] | 1.33 | 0.19 |  | 0.99 |
| L_Basal.nucleus | 404.01 | [ 384 , 424 ] | 396.13 | [ 382 , 410 ] | -7.88 | [ -33 , 17 ] | -0.63 | 0.53 |  | 0.99 |
| L_CA1 | 672.76 | [ 634 , 712 ] | 716.93 | [ 690 , 744 ] | 44.17 | [ -3 , 91 ] | 1.84 | 0.07 |  | 0.99 |
| L_CA3 | 189.42 | [ 176 , 203 ] | 191.01 | [ 181 , 201 ] | 1.59 | [ -15 , 18 ] | 0.19 | 0.85 |  | 0.99 |
| L_CA4 | 255.43 | [ 240 , 271 ] | 271.42 | [ 261 , 282 ] | 15.99 | [ -2 , 34 ] | 1.70 | 0.10 |  | 0.99 |
| L_Central.nucleus | 36.62 | [ 34 , 39 ] | 34.91 | [ 33 , 37 ] | -1.71 | [ -5 , 1 ] | -1.15 | 0.26 |  | 0.99 |
| L_Cortical.nucleus | 20.88 | [ 19 , 23 ] | 20.32 | [ 19 , 22 ] | -0.56 | [ -3 , 2 ] | -0.52 | 0.61 |  | 0.99 |
| L_Corticoamygdaloid.transitio | 191.39 | [ 179 , 204 ] | 191.27 | [ 182 , 200 ] | -0.12 | [ -16 , 15 ] | -0.02 | 0.99 |  | 0.99 |
| L_fimbria | 75.61 | [ 69 , 82 ] | 73.05 | [ 69 , 78 ] | -2.56 | [ -10 , 5 ] | -0.63 | 0.53 |  | 0.99 |
| L_GC.ML.DG | 282.68 | [ 268 , 297 ] | 292.10 | [ 282 , 302 ] | 9.43 | [ -8 , 27 ] | 1.03 | 0.31 |  | 0.99 |
| L_HATA | 62.20 | [ 57 , 67 ] | 63.34 | [ 60 , 67 ] | 1.14 | [ -5 , 7 ] | 0.39 | 0.70 |  | 0.99 |
| L_Hippocampal_tail | 528.30 | [ 495 , 561 ] | 534.79 | [ 512 , 558 ] | 6.48 | [ -33 , 46 ] | 0.32 | 0.75 |  | 0.99 |
| L_hippocampal.fissure | 180.50 | [ 167 , 194 ] | 175.61 | [ 166 , 185 ] | -4.89 | [ -21 , 12 ] | -0.58 | 0.57 |  | 0.99 |
| L_HippoSf | 3653.55 | [ 3471 , 3836 ] | 3672.81 | [ 3547 , 3799 ] | 19.26 | [ -201 , 240 ] | 0.17 | 0.87 |  | 0.99 |
| L_Lateral.nucleus | 601.11 | [ 565 , 637 ] | 587.63 | [ 563 , 613 ] | -13.49 | [ -57 , 30 ] | -0.60 | 0.55 |  | 0.99 |
| L_Medial.nucleus | 15.07 | [ 14 , 17 ] | 14.29 | [ 13 , 15 ] | -0.77 | [ -3 , 1 ] | -0.84 | 0.41 |  | 0.99 |
| L_molecular_layer_HP | 277.59 | [ 257 , 299 ] | 295.60 | [ 281 , 310 ] | 18.02 | [ -8 , 44 ] | 1.38 | 0.17 |  | 0.99 |
| L_Paralaminar.nucleus | 46.56 | [ 44 , 50 ] | 46.30 | [ 44 , 48 ] | -0.26 | [ -4 , 3 ] | -0.14 | 0.89 |  | 0.99 |
| L_parasubiculum | 65.60 | [ 59 , 72 ] | 68.05 | [ 64 , 72 ] | 2.46 | [ -5 , 10 ] | 0.64 | 0.52 |  | 0.99 |
| L_presubiculum | 305.80 | [ 277 , 334 ] | 308.14 | [ 289 , 328 ] | 2.34 | [ -32 , 37 ] | 0.13 | 0.89 |  | 0.99 |
| L_subiculum | 455.88 | [ 418 , 494 ] | 460.86 | [ 434 , 487 ] | 4.97 | [ -42 , 51 ] | 0.21 | 0.84 |  | 0.99 |
| L_Whole_amygdala | 1611.62 | [ 1533 , 1691 ] | 1583.20 | [ 1528 , 1638 ] | -28.42 | [ -124 , 67 ] | -0.58 | 0.56 |  | 0.99 |
| L_Whole_hippocampal_body | 1053.84 | [ 998 , 1110 ] | 1081.88 | [ 1043 , 1121 ] | 28.04 | [ -40 , 96 ] | 0.81 | 0.42 |  | 0.99 |
| L_Whole_hippocampal_head | 1589.14 | [ 1504 , 1674 ] | 1658.64 | [ 1600 , 1718 ] | 69.50 | [ -34 , 173 ] | 1.32 | 0.19 |  | 0.99 |
| L_Whole_hippocampus | 3171.28 | [ 3022 , 3321 ] | 3275.30 | [ 3172 , 3379 ] | 104.02 | [ -77 , 285 ] | 1.13 | 0.27 |  | 0.99 |
| R_Accessory.Basal.nucleus | 284.20 | [ 268 , 300 ] | 280.69 | [ 270 , 292 ] | -3.51 | [ -23 , 16 ] | -0.36 | 0.72 |  | 0.99 |
| R_amygNuc | 3640.43 | [ 3465 , 3816 ] | 3607.25 | [ 3486 , 3729 ] | -33.18 | [ -246 , 180 ] | -0.31 | 0.76 |  | 0.99 |
| R_Anterior.amygdaloid.area.AAA | 62.99 | [ 59 , 67 ] | 60.64 | [ 58 , 63 ] | -2.35 | [ -7 , 2 ] | -0.96 | 0.35 |  | 0.99 |
| R_Basal.nucleus | 437.46 | [ 415 , 460 ] | 415.34 | [ 400 , 431 ] | -22.12 | [ -50 , 5 ] | -1.58 | 0.12 |  | 0.99 |
| R_CA1 | 720.36 | [ 671 , 770 ] | 748.19 | [ 714 , 782 ] | 27.84 | [ -32 , 88 ] | 0.91 | 0.37 |  | 0.99 |
| R_CA3 | 211.43 | [ 199 , 224 ] | 205.27 | [ 197 , 214 ] | -6.16 | [ -21 , 9 ] | -0.81 | 0.42 |  | 0.99 |
| R_CA4 | 272.61 | [ 257 , 288 ] | 273.15 | [ 263 , 284 ] | 0.54 | [ -18 , 19 ] | 0.06 | 0.95 |  | 0.99 |
| R_Central.nucleus | 42.53 | [ 39 , 46 ] | 38.68 | [ 36 , 41 ] | -3.86 | [ -8 , 1 ] | -1.68 | 0.10 |  | 0.99 |
| R_Cortical.nucleus | 23.97 | [ 22 , 26 ] | 23.21 | [ 22 , 24 ] | -0.76 | [ -3 , 1 ] | -0.67 | 0.51 |  | 0.99 |
| R_Corticoamygdaloid.transitio | 210.98 | [ 195 , 227 ] | 205.68 | [ 195 , 217 ] | -5.30 | [ -25 , 14 ] | -0.54 | 0.60 |  | 0.99 |
| R_fimbria | 80.05 | [ 72 , 88 ] | 78.55 | [ 73 , 84 ] | -1.49 | [ -11 , 8 ] | -0.32 | 0.75 |  | 0.99 |
| R_GC.ML.DG | 286.51 | [ 273 , 301 ] | 282.28 | [ 273 , 292 ] | -4.23 | [ -21 , 13 ] | -0.49 | 0.63 |  | 0.99 |
| R_HATA | 66.52 | [ 59 , 74 ] | 67.27 | [ 62 , 72 ] | 0.75 | [ -8 , 10 ] | 0.17 | 0.87 |  | 0.99 |
| R_Hippocampal_tail | 483.94 | [ 449 , 519 ] | 490.93 | [ 467 , 515 ] | 7.00 | [ -35 , 49 ] | 0.33 | 0.75 |  | 0.99 |
| R_hippocampal.fissure | 155.76 | [ 145 , 167 ] | 147.54 | [ 140 , 155 ] | -8.23 | [ -22 , 5 ] | -1.21 | 0.24 |  | 0.99 |
| R_HippoSf | 3640.43 | [ 3465 , 3816 ] | 3607.25 | [ 3486 , 3729 ] | -33.18 | [ -246 , 180 ] | -0.31 | 0.76 |  | 0.99 |
| R_Lateral.nucleus | 638.04 | [ 604 , 673 ] | 629.69 | [ 606 , 654 ] | -8.35 | [ -50 , 33 ] | -0.39 | 0.70 |  | 0.99 |
| R_Medial.nucleus | 20.74 | [ 19 , 23 ] | 17.16 | [ 16 , 19 ] | -3.58 | [ -6 , -1 ] | -2.81 | 0.01 |  | 0.42 |
| R_molecular_layer_HP | 264.07 | [ 247 , 281 ] | 262.43 | [ 251 , 274 ] | -1.64 | [ -22 , 19 ] | -0.16 | 0.88 |  | 0.99 |
| R_Paralaminar.nucleus | 44.41 | [ 41 , 47 ] | 40.92 | [ 39 , 43 ] | -3.49 | [ -7 , 0 ] | -1.85 | 0.07 |  | 0.99 |
| R_parasubiculum | 68.33 | [ 62 , 74 ] | 65.18 | [ 61 , 69 ] | -3.15 | [ -10 , 4 ] | -0.87 | 0.39 |  | 0.99 |
| R_presubiculum | 270.59 | [ 247 , 294 ] | 258.46 | [ 242 , 274 ] | -12.13 | [ -40 , 16 ] | -0.85 | 0.40 |  | 0.99 |
| R_subiculum | 414.20 | [ 387 , 441 ] | 403.46 | [ 385 , 422 ] | -10.74 | [ -44 , 22 ] | -0.64 | 0.53 |  | 0.99 |
| R_Whole_amygdala | 1765.32 | [ 1680 , 1851 ] | 1712.00 | [ 1653 , 1771 ] | -53.32 | [ -157 , 50 ] | -1.01 | 0.32 |  | 0.99 |
| R_Whole_hippocampal_body | 1024.22 | [ 976 , 1072 ] | 1016.31 | [ 983 , 1049 ] | -7.91 | [ -66 , 50 ] | -0.27 | 0.79 |  | 0.99 |
| R_Whole_hippocampal_head | 1630.45 | [ 1538 , 1723 ] | 1627.94 | [ 1564 , 1692 ] | -2.51 | [ -115 , 110 ] | -0.04 | 0.97 |  | 0.99 |
| R_Whole_hippocampus | 3138.60 | [ 2998 , 3279 ] | 3135.18 | [ 3038 , 3233 ] | -3.42 | [ -174 , 167 ] | -0.04 | 0.97 |  | 0.99 |

Abbreviations: CA: Cornu Ammonis; GC: granule cell; ML: molecular layer; DG: dentate gyrus; HP: hippocampus; HATA: hippocampal amygdala transition area

Supplementary Table S6. Subfield volume differences between ketamine and placebo scans for acute and interim scans between healthy volunteer (HV) and treatment-resistant depression (TRD) groups at 3T.

| **Region** | **Interval** | **estimate** | **est.CI** | **t.ratio** | **p.value** | **p.adj** |
| --- | --- | --- | --- | --- | --- | --- |
| L_Lateral.nucleus | Acute | -7.44 | [ -23 , 9 ] | -0.91 | 0.36 | 0.99 |
| L_Lateral.nucleus | Interim | -7.38 | [ -22 , 8 ] | -0.97 | 0.34 | 0.99 |
| L_Basal.nucleus | Acute | -10.8 | [ -24 , 3 ] | -1.56 | 0.12 | 0.99 |
| L_Basal.nucleus | Interim | -0.61 | [ -13 , 12 ] | -0.09 | 0.93 | 0.99 |
| L_Accessory.Basal.nucleus | Acute | -8.23 | [ -19 , 3 ] | -1.46 | 0.15 | 0.99 |
| L_Accessory.Basal.nucleus | Interim | -2.09 | [ -12 , 8 ] | -0.39 | 0.69 | 0.99 |
| L_Anterior.amygdaloid.area.AAA | Acute | 0.52 | [ -3 , 4 ] | 0.32 | 0.75 | 0.99 |
| L_Anterior.amygdaloid.area.AAA | Interim | 3.79 | [ 1 , 7 ] | 2.46 | 0.02 | 0.99 |
| L_Central.nucleus | Acute | -1.92 | [ -6 , 2 ] | -1.01 | 0.31 | 0.99 |
| L_Central.nucleus | Interim | -0.96 | [ -4 , 3 ] | -0.54 | 0.59 | 0.99 |
| L_Medial.nucleus | Acute | 0.21 | [ -3 , 3 ] | 0.15 | 0.88 | 0.99 |
| L_Medial.nucleus | Interim | -1.39 | [ -4 , 1 ] | -1.03 | 0.3 | 0.99 |
| L_Cortical.nucleus | Acute | -1.2 | [ -3 , 1 ] | -1.29 | 0.2 | 0.99 |
| L_Cortical.nucleus | Interim | -1.25 | [ -3 , 0 ] | -1.43 | 0.16 | 0.99 |
| L_Corticoamygdaloid.transitio | Acute | -0.14 | [ -5 , 5 ] | -0.05 | 0.96 | 0.99 |
| L_Corticoamygdaloid.transitio | Interim | -1.25 | [ -6 , 4 ] | -0.5 | 0.62 | 0.99 |
| L_Paralaminar.nucleus | Acute | -1.39 | [ -3 , 0 ] | -1.54 | 0.13 | 0.99 |
| L_Paralaminar.nucleus | Interim | -1.06 | [ -3 , 1 ] | -1.25 | 0.21 | 0.99 |
| L_Whole_amygdala | Acute | -30.77 | [ -77 , 15 ] | -1.31 | 0.19 | 0.99 |
| L_Whole_amygdala | Interim | -12.06 | [ -55 , 31 ] | -0.55 | 0.59 | 0.99 |
| L_amygNuc | Acute | 94.98 | [ 30 , 160 ] | 2.88 | 0.01 | 0.53 |
| L_amygNuc | Interim | 47.98 | [ -13 , 109 ] | 1.55 | 0.12 | 0.99 |
| L_Hippocampal_tail | Acute | -7.51 | [ -25 , 10 ] | -0.84 | 0.4 | 0.99 |
| L_Hippocampal_tail | Interim | 3.63 | [ -13 , 20 ] | 0.43 | 0.67 | 0.99 |
| L_hippocampal.fissure | Acute | -6.03 | [ -21 , 9 ] | -0.77 | 0.44 | 0.99 |
| L_hippocampal.fissure | Interim | 0.15 | [ -14 , 14 ] | 0.02 | 0.98 | 0.99 |
| L_parasubiculum | Acute | 0.19 | [ -3 , 3 ] | 0.12 | 0.91 | 0.99 |
| L_parasubiculum | Interim | 2.06 | [ -1 , 5 ] | 1.37 | 0.17 | 0.99 |
| L_fimbria | Acute | -1.41 | [ -8 , 5 ] | -0.42 | 0.68 | 0.99 |
| L_fimbria | Interim | -2.14 | [ -8 , 4 ] | -0.67 | 0.5 | 0.99 |
| L_HATA | Acute | -1.84 | [ -5 , 1 ] | -1.17 | 0.25 | 0.99 |
| L_HATA | Interim | 1.21 | [ -2 , 4 ] | 0.82 | 0.41 | 0.99 |
| L_Whole_hippocampal_body | Acute | -4.31 | [ -30 , 21 ] | -0.33 | 0.74 | 0.99 |
| L_Whole_hippocampal_body | Interim | 13.34 | [ -11 , 38 ] | 1.08 | 0.28 | 0.99 |
| L_Whole_hippocampal_head | Acute | -19.53 | [ -46 , 7 ] | -1.42 | 0.16 | 0.99 |
| L_Whole_hippocampal_head | Interim | 19.11 | [ -6 , 44 ] | 1.48 | 0.14 | 0.99 |
| L_Whole_hippocampus | Acute | -28.43 | [ -77 , 20 ] | -1.15 | 0.25 | 0.99 |
| L_Whole_hippocampus | Interim | 35.23 | [ -10 , 81 ] | 1.52 | 0.13 | 0.99 |
| L_HippoSf | Acute | 94.98 | [ 30 , 160 ] | 2.88 | 0.01 | 0.53 |
| L_HippoSf | Interim | 47.98 | [ -13 , 109 ] | 1.55 | 0.12 | 0.99 |
| L_CA1 | Acute | -4.86 | [ -19 , 9 ] | -0.69 | 0.49 | 0.99 |
| L_CA1 | Interim | 2.94 | [ -10 , 16 ] | 0.45 | 0.66 | 0.99 |
| L_CA3 | Acute | 0.54 | [ -6 , 7 ] | 0.17 | 0.87 | 0.99 |
| L_CA3 | Interim | 5.25 | [ -1 , 11 ] | 1.72 | 0.09 | 0.99 |
| L_CA4 | Acute | -0.62 | [ -6 , 5 ] | -0.21 | 0.83 | 0.99 |
| L_CA4 | Interim | 5.66 | [ 0 , 11 ] | 2.03 | 0.04 | 0.99 |
| L_subiculum | Acute | -9.24 | [ -19 , 0 ] | -1.91 | 0.06 | 0.99 |
| L_subiculum | Interim | 1.47 | [ -7 , 10 ] | 0.32 | 0.75 | 0.99 |
| L_presubiculum | Acute | 2.84 | [ -8 , 14 ] | 0.5 | 0.62 | 0.99 |
| L_presubiculum | Interim | 3.94 | [ -6 , 14 ] | 0.74 | 0.46 | 0.99 |
| L_GC.ML.DG | Acute | -1.63 | [ -8 , 4 ] | -0.53 | 0.59 | 0.99 |
| L_GC.ML.DG | Interim | 6.18 | [ 1 , 12 ] | 2.15 | 0.03 | 0.99 |
| L_molecular_layer_HP | Acute | -4.28 | [ -14 , 5 ] | -0.88 | 0.38 | 0.99 |
| L_molecular_layer_HP | Interim | 5.23 | [ -4 , 14 ] | 1.15 | 0.26 | 0.99 |
| R_Lateral.nucleus | Acute | 4.63 | [ -16 , 25 ] | 0.45 | 0.66 | 0.99 |
| R_Lateral.nucleus | Interim | -7.69 | [ -27 , 11 ] | -0.79 | 0.43 | 0.99 |
| R_Basal.nucleus | Acute | 2.2 | [ -11 , 16 ] | 0.32 | 0.75 | 0.99 |
| R_Basal.nucleus | Interim | 0.98 | [ -12 , 14 ] | 0.15 | 0.88 | 0.99 |
| R_Accessory.Basal.nucleus | Acute | 3.28 | [ -8 , 14 ] | 0.58 | 0.57 | 0.99 |
| R_Accessory.Basal.nucleus | Interim | 3.65 | [ -7 , 14 ] | 0.68 | 0.5 | 0.99 |
| R_Anterior.amygdaloid.area.AAA | Acute | 0.71 | [ -3 , 5 ] | 0.35 | 0.73 | 0.99 |
| R_Anterior.amygdaloid.area.AAA | Interim | -0.26 | [ -4 , 3 ] | -0.13 | 0.89 | 0.99 |
| R_Central.nucleus | Acute | 1.15 | [ -3 , 5 ] | 0.57 | 0.57 | 0.99 |
| R_Central.nucleus | Interim | -0.88 | [ -5 , 3 ] | -0.46 | 0.65 | 0.99 |
| R_Medial.nucleus | Acute | 0.38 | [ -2 , 3 ] | 0.27 | 0.79 | 0.99 |
| R_Medial.nucleus | Interim | -0.6 | [ -3 , 2 ] | -0.45 | 0.66 | 0.99 |
| R_Cortical.nucleus | Acute | -0.36 | [ -2 , 1 ] | -0.41 | 0.69 | 0.99 |
| R_Cortical.nucleus | Interim | -0.01 | [ -2 , 2 ] | -0.01 | 0.99 | 0.99 |
| R_Corticoamygdaloid.transitio | Acute | 2.74 | [ -2 , 8 ] | 1.12 | 0.27 | 0.99 |
| R_Corticoamygdaloid.transitio | Interim | 3.4 | [ -1 , 8 ] | 1.47 | 0.14 | 0.99 |
| R_Paralaminar.nucleus | Acute | -0.11 | [ -2 , 1 ] | -0.13 | 0.9 | 0.99 |
| R_Paralaminar.nucleus | Interim | -0.33 | [ -2 , 1 ] | -0.43 | 0.67 | 0.99 |
| R_Whole_amygdala | Acute | 14.06 | [ -36 , 64 ] | 0.55 | 0.59 | 0.99 |
| R_Whole_amygdala | Interim | -1.66 | [ -49 , 45 ] | -0.07 | 0.95 | 0.99 |
| R_amygNuc | Acute | 28.29 | [ -39 , 95 ] | 0.83 | 0.41 | 0.99 |
| R_amygNuc | Interim | 46.58 | [ -16 , 110 ] | 1.45 | 0.15 | 0.99 |
| R_Hippocampal_tail | Acute | -2.43 | [ -17 , 12 ] | -0.32 | 0.75 | 0.99 |
| R_Hippocampal_tail | Interim | -5.64 | [ -20 , 8 ] | -0.79 | 0.43 | 0.99 |
| R_hippocampal.fissure | Acute | -2.59 | [ -16 , 11 ] | -0.39 | 0.7 | 0.99 |
| R_hippocampal.fissure | Interim | -23.98 | [ -36 , -12 ] | -3.8 | 0 | 0.03 |
| R_parasubiculum | Acute | 3.95 | [ 1 , 7 ] | 2.76 | 0.01 | 0.74 |
| R_parasubiculum | Interim | -0.09 | [ -3 , 3 ] | -0.06 | 0.95 | 0.99 |
| R_fimbria | Acute | -3.29 | [ -9 , 3 ] | -1.11 | 0.27 | 0.99 |
| R_fimbria | Interim | -4.47 | [ -10 , 1 ] | -1.61 | 0.11 | 0.99 |
| R_HATA | Acute | 1.44 | [ -2 , 4 ] | 0.95 | 0.35 | 0.99 |
| R_HATA | Interim | 3.1 | [ 0 , 6 ] | 2.17 | 0.03 | 0.99 |
| R_Whole_hippocampal_body | Acute | 1.74 | [ -21 , 24 ] | 0.15 | 0.88 | 0.99 |
| R_Whole_hippocampal_body | Interim | 6.35 | [ -15 , 27 ] | 0.6 | 0.55 | 0.99 |
| R_Whole_hippocampal_head | Acute | 9.01 | [ -24 , 42 ] | 0.54 | 0.59 | 0.99 |
| R_Whole_hippocampal_head | Interim | 33.92 | [ 3 , 65 ] | 2.15 | 0.03 | 0.99 |
| R_Whole_hippocampus | Acute | 8.41 | [ -44 , 61 ] | 0.31 | 0.76 | 0.99 |
| R_Whole_hippocampus | Interim | 34.67 | [ -15 , 84 ] | 1.38 | 0.17 | 0.99 |
| R_HippoSf | Acute | 28.29 | [ -39 , 95 ] | 0.83 | 0.41 | 0.99 |
| R_HippoSf | Interim | 46.58 | [ -16 , 110 ] | 1.45 | 0.15 | 0.99 |
| R_CA1 | Acute | 3.81 | [ -11 , 19 ] | 0.5 | 0.62 | 0.99 |
| R_CA1 | Interim | 18.4 | [ 4 , 32 ] | 2.56 | 0.01 | 0.99 |
| R_CA3 | Acute | -2.17 | [ -8 , 3 ] | -0.77 | 0.45 | 0.99 |
| R_CA3 | Interim | 2.23 | [ -3 , 7 ] | 0.84 | 0.4 | 0.99 |
| R_CA4 | Acute | -0.58 | [ -7 , 6 ] | -0.18 | 0.86 | 0.99 |
| R_CA4 | Interim | 3.95 | [ -2 , 10 ] | 1.32 | 0.19 | 0.99 |
| R_subiculum | Acute | 1.45 | [ -8 , 11 ] | 0.3 | 0.77 | 0.99 |
| R_subiculum | Interim | -3.23 | [ -12 , 6 ] | -0.7 | 0.48 | 0.99 |
| R_presubiculum | Acute | 7 | [ -4 , 18 ] | 1.22 | 0.23 | 0.99 |
| R_presubiculum | Interim | 6.13 | [ -4 , 17 ] | 1.13 | 0.26 | 0.99 |
| R_GC.ML.DG | Acute | 0.21 | [ -6 , 6 ] | 0.07 | 0.95 | 0.99 |
| R_GC.ML.DG | Interim | 4.25 | [ -2 , 10 ] | 1.44 | 0.15 | 0.99 |
| R_molecular_layer_HP | Acute | -0.31 | [ -11 , 10 ] | -0.06 | 0.95 | 0.99 |
| R_molecular_layer_HP | Interim | 10.43 | [ 1 , 20 ] | 2.13 | 0.04 | 0.99 |

Abbreviations: CA: Cornu Ammonis; GC: granule cell; ML: molecular layer; DG: dentate gyrus; HP: hippocampus; HATA: hippocampal amygdala transition area; CI: Confidence interval

Supplementary Table S7. Percent change in volume from acute-ketamine to baseline scan

| **Subfield** | **3T.Mean** | | **3T.CI** | | **7T.Mean** | | **7T.CI** |  |
| --- | --- | --- | --- | --- | --- | --- | --- | --- |
| CA1 | -0.44 | | [ -2 , 1 ] | | 0.43 | | [ 0 , 1 ] |  |
| CA3 | -0.53 | | [ -2 , 1 ] | | 0.57 | | [ -0 , 1 ] |  |
| CA4 | 0.26 | | [ -1 , 1 ] | | 0.42 | | [ -0 , 1 ] |  |
| fimbria | -0.16 | | [ -3 , 2 ] | | 1.08 | | [ -0 , 2 ] |  |
| GC.ML.DG | 0.18 | | [ -2 , 3 ] | | 0.27 | | [ -0 , 1 ] |  |
| HATA | 1.46 | | [ -0 , 3 ] | | 0.09 | | [ -1 , 1 ] |  |
| Hippocampal_tail | 0.74 | | [ -0 , 1 ] | | 0.54 | | [ -0 , 1 ] |  |
| hippocampal.fissure | 1.77 | | [ -2 , 6 ] | | -1.32 | | [ -2 , -0 ] |  |
| molecular_layer_HP | -0.28 | | [ -2 , 1 ] | | 1.42 | | [ 0 , 2 ] |  |
| parasubiculum | 1.67 | | [ 1 , 3 ] | | -0.25 | | [ -1 , 1 ] |  |
| presubiculum | -0.51 | | [ -2 , 1 ] | | 0.08 | | [ -1 , 1 ] |  |
| subiculum | -0.23 | | [ -2 , 1 ] | | 0.11 | | [ -0 , 1 ] |  |
| Whole_hippocampal_body | -0.47 | | [ -2 , 1 ] | | 0.55 | | [ 0 , 1 ] |  |
| Whole_hippocampal_head | 0.02 | | [ -2 , 3 ] | | 0.33 | | [ 0 , 1 ] |  |
| Whole_hippocampus | -0.01 | | [ -3 , 2 ] | | 0.44 | | [ 0 , 1 ] |  |
| Accessory.Basal.nucleus | 2.51 | | [ 1 , 4 ] | | -0.62 | | [ -1 , 0 ] |  |
| Anterior.amygdaloid.area.AAA | 0.24 | | [ -1 , 2 ] | | -0.63 | | [ -2 , 0 ] |  |
| Basal.nucleus | 2.15 | | [ 1 , 3 ] | | -0.32 | | [ -1 , 0 ] |  |
| Central.nucleus | 4.20 | | [ 2 , 7 ] | | -1.59 | | [ -3 , -0 ] |  |
| Cortical.nucleus | 2.51 | | [ 0 , 5 ] | | -0.35 | | [ -1 , 1 ] |  |
| Corticoamygdaloid.transitio | 2.61 | | [ 1 , 4 ] | | 0.24 | | [ -0 , 1 ] |  |
| Lateral.nucleus | 1.12 | | [ 0 , 2 ] | | 0.07 | | [ -0 , 1 ] |  |
| Medial.nucleus | 6.69 | | [ 2 , 11 ] | | -1.46 | | [ -3 , 0 ] |  |
| Paralaminar.nucleus | 2.32 | | [ 1 , 4 ] | | -0.35 | | [ -1 , 0 ] |  |
| Whole_amygdala | 1.88 | [ 1 , 3 ] | | -0.19 | | [ -1 , 0 ] | | |

Abbreviations: CA: Cornu Ammonis; GC: granule cell; ML: molecular layer; DG: dentate gyrus; HP: hippocampus; HATA: hippocampal amygdala transition area, CI: Confidence interval

Supplementary Table S8. Subfield dice and intraclass correlation coefficients (ICCs)

| **roi** | **Dice.3T** | **[CI]** | **Dice.7T** | **[CI]** | **ICC.3T** | **ICC.3T.CI** | **ICC.7T** | **ICC.7T.CI** |
| --- | --- | --- | --- | --- | --- | --- | --- | --- |
| Accessory.Basal.nucleus | 0.91 | [ 0.85 , 0.96 ] | 0.92 | [ 0.87 , 0.95 ] | 0.99 | [ 0.99 , 0.99 ] | 0.99 | [ 0.99 , 0.99 ] |
| Anterior.amygdaloid.area | NA | [ 0.00 , 0.88 ] | 0.77 | [ 0.44 , 0.92 ] | 0.99 | [ 0.99 , 0.99 ] | 0.99 | [ 0.99 , 0.99 ] |
| Basal.nucleus | 0.92 | [ 0.86 , 0.96 ] | 0.93 | [ 0.89 , 0.96 ] | 0.99 | [ 0.99 , 0.99 ] | 0.99 | [ 0.99 , 0.99 ] |
| CA1 | 0.83 | [ 0.78 , 0.89 ] | 0.89 | [ 0.85 , 0.92 ] | 1.00 | [ 1.00 , 1.00 ] | 1.00 | [ 1.00 , 1.00 ] |
| CA3 | 0.73 | [ 0.57 , 0.83 ] | 0.79 | [ 0.71 , 0.86 ] | 1.00 | [ 1.00 , 1.00 ] | 1.00 | [ 1.00 , 1.00 ] |
| CA4 | 0.86 | [ 0.75 , 0.92 ] | 0.91 | [ 0.87 , 0.94 ] | 1.00 | [ 1.00 , 1.00 ] | 1.00 | [ 1.00 , 1.00 ] |
| Central.nucleus | 0.59 | [ 0.34 , 0.75 ] | 0.60 | [ 0.37 , 0.77 ] | 0.98 | [ 0.97 , 0.98 ] | 0.98 | [ 0.97 , 0.98 ] |
| Cortical.nucleus | 0.58 | [ 0.28 , 0.76 ] | 0.65 | [ 0.40 , 0.79 ] | 0.98 | [ 0.97 , 0.98 ] | 0.98 | [ 0.97 , 0.98 ] |
| Corticoamygdaloid.transition | 0.79 | [ 0.65 , 0.89 ] | 0.82 | [ 0.73 , 0.90 ] | 0.99 | [ 0.99 , 0.99 ] | 0.99 | [ 0.99 , 0.99 ] |
| fimbria | 0.70 | [ 0.53 , 0.84 ] | 0.78 | [ 0.64 , 0.87 ] | 0.99 | [ 0.98 , 0.99 ] | 0.99 | [ 0.98 , 0.99 ] |
| HATA | 0.77 | [ 0.68 , 0.86 ] | 0.79 | [ 0.68 , 0.87 ] | 0.99 | [ 0.99 , 0.99 ] | 0.99 | [ 0.99 , 0.99 ] |
| Lateral.nucleus | 0.92 | [ 0.86 , 0.95 ] | 0.94 | [ 0.91 , 0.96 ] | 1.00 | [ 1.00 , 1.00 ] | 1.00 | [ 1.00 , 1.00 ] |
| Medial.nucleus | 0.29 | [ 0.05 , 0.57 ] | 0.37 | [ 0.08 , 0.60 ] | 0.96 | [ 0.95 , 0.97 ] | 0.96 | [ 0.95 , 0.97 ] |
| molecular_layer_HP | 0.74 | [ 0.62 , 0.82 ] | 0.58 | [ 0.44 , 0.70 ] | 1.00 | [ 1.00 , 1.00 ] | 1.00 | [ 1.00 , 1.00 ] |
| Paralaminar.nucleus | 0.55 | [ 0.32 , 0.73 ] | NA | [ 0.42 , 0.81 ] | 0.99 | [ 0.99 , 0.99 ] | 0.99 | [ 0.99 , 0.99 ] |
| parasubiculum | 0.73 | [ 0.55 , 0.85 ] | 0.81 | [ 0.70 , 0.88 ] | 0.99 | [ 0.99 , 0.99 ] | 0.99 | [ 0.99 , 0.99 ] |
| presubiculum | 0.83 | [ 0.72 , 0.91 ] | 0.87 | [ 0.81 , 0.91 ] | 1.00 | [ 0.99 , 1.00 ] | 1.00 | [ 0.99 , 1.00 ] |
| subiculum | 0.85 | [ 0.75 , 0.91 ] | 0.91 | [ 0.87 , 0.93 ] | 1.00 | [ 1.00 , 1.00 ] | 1.00 | [ 1.00 , 1.00 ] |

Abbreviations: CA: Cornu Ammonis; GC: granule cell; ML: molecular layer; DG: dentate gyrus; HP: hippocampus; HATA: hippocampal amygdala transition area; CI: Confidence interval; ICC: intraclass correlation coefficient.
